# Common and unique elements of the ABA-regulated transcriptome of Arabidopsis guard cells

**DOI:** 10.1186/1471-2164-12-216

**Published:** 2011-05-09

**Authors:** Rui-Sheng Wang, Sona Pandey, Song Li, Timothy E Gookin, Zhixin Zhao, Réka Albert, Sarah M Assmann

**Affiliations:** 1 Department of Physics, Pennsylvania State University, University Park, PA 16802, USA; 2 Department of Biology, Pennsylvania State University, University Park, PA 16802, USA

## Abstract

**Background:**

In the presence of drought and other desiccating stresses, plants synthesize and redistribute the phytohormone abscisic acid (ABA). ABA promotes plant water conservation by acting on specialized cells in the leaf epidermis, guard cells, which border and regulate the apertures of stomatal pores through which transpirational water loss occurs. Following ABA exposure, solute uptake into guard cells is rapidly inhibited and solute loss is promoted, resulting in inhibition of stomatal opening and promotion of stomatal closure, with consequent plant water conservation. There is a wealth of information on the guard cell signaling mechanisms underlying these rapid ABA responses. To investigate ABA regulation of gene expression in guard cells in a systematic genome-wide manner, we analyzed data from global transcriptomes of guard cells generated with Affymetrix ATH1 microarrays, and compared these results to ABA regulation of gene expression in leaves and other tissues.

**Results:**

The 1173 ABA-regulated genes of guard cells identified by our study share significant overlap with ABA-regulated genes of other tissues, and are associated with well-defined ABA-related promoter motifs such as ABREs and DREs. However, we also computationally identified a unique *cis*-acting motif, GTCGG, associated with ABA-induction of gene expression specifically in guard cells. In addition, approximately 300 genes showing ABA-regulation unique to this cell type were newly uncovered by our study. Within the ABA-regulated gene set of guard cells, we found that many of the genes known to encode ion transporters associated with stomatal opening are down-regulated by ABA, providing one mechanism for long-term maintenance of stomatal closure during drought. We also found examples of both negative and positive feedback in the transcriptional regulation by ABA of known ABA-signaling genes, particularly with regard to the PYR/PYL/RCAR class of soluble ABA receptors and their downstream targets, the type 2C protein phosphatases. Our data also provide evidence for cross-talk at the transcriptional level between ABA and another hormonal inhibitor of stomatal opening, methyl jasmonate.

**Conclusions:**

Our results engender new insights into the basic cell biology of guard cells, reveal common and unique elements of ABA-regulation of gene expression in guard cells, and set the stage for targeted biotechnological manipulations to improve plant water use efficiency.

## Background

Drought stress imposes one of the greatest limitations to crop growth and yield worldwide [1]. Limitations in fresh water availability are predicted to become an increasing problem due to industrialization and continuing global climate change [2-4]. One of the central mechanisms in plant drought tolerance is a reduction in stomatal apertures at the leaf surface, with consequent increase in water use efficiency [5-7]. Abscisic acid (ABA) is a major plant hormone which inhibits growth and promotes tolerance of abiotic stresses such as drought, salinity and cold [8-10]. Under drought conditions, plants synthesize and redistribute ABA which triggers cellular responses in guard cells, specialized cells that flank the stomatal pores, leading to inhibition of stomatal opening and promotion of stomatal closure and thereby reducing plant water loss. Thus, an improved understanding of guard cell responses to ABA is relevant for development of cultivars with improved productivity under drought conditions.

Guard cells also have become an advanced model system for understanding plant signal transduction [11-13]. Much of the research on guard cell responses has focused on the signaling cascades underlying rapid responses to ABA as well as to other inputs such as light and CO_2 _[14]. However, in addition to these post-transcriptional responses, guard cells also respond to ABA at the level of the transcriptome. For example, Li *et al. *showed in *Vicia faba *guard cell protoplasts that these cells respond with an increase in dehydrin transcript levels after just 15 min. of ABA exposure [15], suggesting that some of the cellular signaling processes that are observed could be influenced by transcriptional events. Leonhardt *et al. *published an expression analysis of the ABA-related transcriptome of guard cell protoplasts [16], utilizing the Affymetrix AG GeneChip, which represents ~8200 Arabidopsis genes. In those experiments, whole plants were treated with 100 μM ABA for four hours, and transcriptional inhibitors were applied during the protoplasting process, with the idea of stabilizing transcript levels. The authors demonstrated ABA-regulation of approximately 150 genes in guard cell protoplasts. To extend the investigation of gene expression in guard cells, Yang *et al. *isolated guard cell promoter candidates based on 4 Arabidopsis guard cell microarrays from 4 different treatments using whole-genome Affymetrix ATH1 chips [17], which profile ~24,000 genes.

To date, however, there has been no systematic replicated study that investigates ABA regulation of gene expression in guard cells in a genome-wide manner. A comprehensive analysis of the global ABA-related transcriptome of guard cells and its relationship to known guard-cell signaling cascades promises to engender new insights into the basic cell biology of guard cells. In addition, the wealth of microarray data regarding ABA-regulated transcriptomes of other tissues, including whole plants [18,19], seedlings [20-26], and seeds [27], provides an opportunity to assess the extent to which the specialized guard cells employ unique vs. common promoter motifs and unique vs. common gene targets in ABA regulation of gene expression. Given growing evidence of hormonal cross-talk in physiological responses [10], another interesting question is whether or not ABA-regulated transcripts of guard cells are regulated by other hormones in other plant tissues. Finally, manipulation of transcriptional regulation of key genes is one of the most common approaches utilized in applied research aimed at improving plant stress tolerance [28-30], so genome-wide analysis of the Arabidopsis guard cell transcriptome may yield candidate genes for translational research. In this study, we performed a systematic microarray analysis of gene expression profile data from global transcriptomes of guard cells generated with Affymetrix ATH1 chips to explore the above topics.

## Results

### Identification of ABA-responsive genes in guard cells and leaves

**Figure 1:**
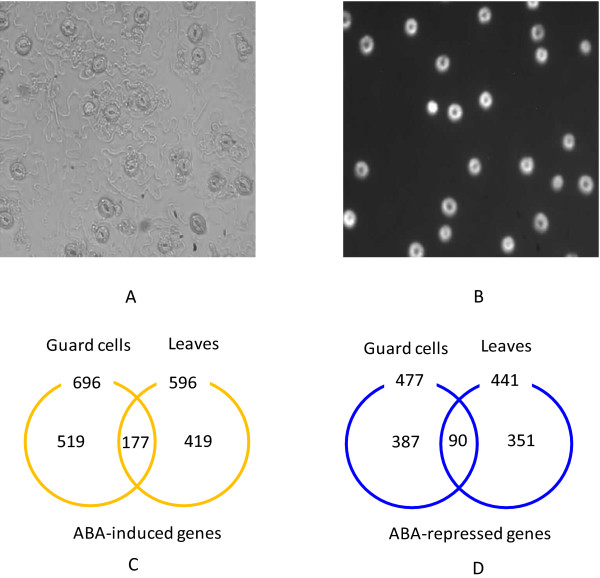
**Images of guard cells and overview of guard cell ABA-regulated genes and leaf ABA-regulated genes**. (A) Bright-field image: Epidermal peel with intact guard cells after partial cell wall digestion. (B) Fluorescent image: Vital staining of epidermal peels with FDA reveals that guard cells are the only intact cell type. (C) Overlap of guard cell ABA-induced genes and leaf ABA-induced genes. (D) Overlap of guard cell ABA-repressed genes and leaf ABA-repressed genes.

We and others [16] have observed that when guard cell protoplasts were used as the source of RNA, stress-induced genes could be induced by protoplasting, even without ABA treatment. Therefore, we employed a protocol [31] whereby isolated epidermal peels with guard cells as the only intact cell type (Figure 1A and Figure 1B) were used as the source of guard cell RNA for microarray analysis.

For each tissue by treatment combination (guard cell vs. leaf; ABA vs. ethanol as solvent control), three independent biological samples were assessed using Affymetrix whole genome ATH1 chips [31]. For the present study, to reliably identify ABA-responsive genes, we adopted two different methods - a Boolean method [31] and a standard linear model method [32] - and focused on the set of ABA-regulated genes jointly confirmed by both approaches (see Methods for details). Through this overlapping analysis we identified 1173 ABA-responsive genes in Col (Arabidopsis Col-0) guard cells (696 ABA-induced genes and 477 ABA-repressed genes) and 1037 ABA-responsive genes in Col leaves (596 ABA-induced genes and 441 ABA-repressed genes). The ABA-regulated genes in guard cells and leaves are listed in Additional file 1 and Additional file 2, respectively. As shown in Figure 1C and 1D, among all the ABA-induced genes, only 177 genes are ABA-induced in both guard cells and leaves, while the rest show differential regulation with respect to tissue type. The overlap of guard cell ABA-repressed genes and leaf ABA-repressed genes is even smaller with only 90 genes common to both guard cells and leaves. There are only 4 genes that are oppositely regulated by ABA in guard cells vs. leaves: AT5G20840, *EXL3 *(AT5G51550), and *CIL *(AT4G25990) are induced by ABA in guard cells but repressed by ABA in leaves, and *KCS5 *(AT1G25450) is induced by ABA in leaves but repressed by ABA in guard cells.

### Validation of ABA-responsive genes from microarray analysis by Q-PCR

**Figure 2:**
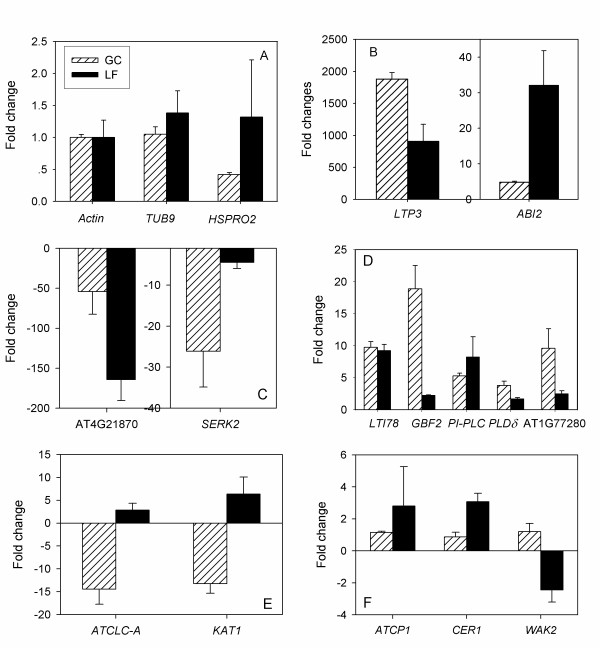
**Validation of a set of ABA responsive genes identified from microarray analysis by Q-PCR**. GC: guard cells. LF: leaves. (A) Expression fold changes of the reference gene *Actin *and two control genes in response to ABA. (B) Expression fold changes of genes that are up-regulated by ABA in both leaf and guard cell microarrays. (C) Expression fold changes of genes that are down-regulated by ABA in both leaf and guard cell microarrays. (D) Expression fold changes of genes that are up-regulated by ABA in guard cell microarrays only. (E) Expression fold changes of genes that are down-regulated by ABA in guard cell microarrays only. (F) Expression fold changes of genes that are up-regulated by ABA (*ATCP1 *and *CER1*) and down-regulated by ABA (*WAK2*) in leaf microarrays only. See text for specific gene locus identifiers.

To evaluate the reliability of ABA-regulation derived from the microarray data and validate identified ABA-responsive genes, we performed Q-PCR experiments on 3 genes identified as not responsive to ABA and 14 genes that showed differential expression in response to ABA in either guard cells, leaves, or both tissues by our microarray analysis (Figure 2). The three negative control genes were *Actin *(*Actin 2*: AT3G18780 and *Actin 8*: AT1G49240), a *β*-tubulin like protein *TUB9 *(AT4G20890), and a putative nematode resistant protein Arabidopsis ortholog of sugar beet HS1 PRO-1 2 (*HSPRO2*, AT2G40000). Among the ABA-responsive genes, we chose two genes that exhibited ABA-upregulation in both guard cells and leaves in our microarray analysis: *ABI2 *(AT5G57050), which encodes one of several PP2C-type protein phosphatases that negatively regulate many aspects of ABA signaling, including ABA-induced stomatal closure [33-35], and the non-specific lipid transfer protein 3 (*LTP3*, AT5G59320), which is a drought-responsive gene [36]. In addition, we analyzed two genes that showed ABA-repression in both guard cell and leaf microarrays: a heat shock protein (AT4G21870) and a somatic embryogenesis receptor-like kinase 2 (*SERK2*, AT1G34210). For the category of ABA-induction in guard cell but not leaf microarrays, five genes were chosen for Q-PCR evaluation: *LTI78/RD29A *(AT5G52310), identified as a cold and salt stress-induced gene in a leaf transcription study [37,38], two genes encoding phospholipases known to be involved in ABA signaling, namely a phospholipase C gene, *PI-PLC *(AT3G55940) and a *PLDδ *homolog (AT4G35790), as well as *GBF2 *(AT4G01120), and a protein kinase (AT1G77280). For genes that showed ABA-repression in guard cell but not leaf microarrays, we performed Q-PCR assessment of a vacuolar anion channel gene *ATCLC-A *(AT5G40890) and the ion channel gene *KAT1 *(AT5G46240), which mediates K^+ ^uptake during stomatal opening. For genes that were ABA-responsive only in the leaf microarrays, we tested *ECERIFERUM 1 *(*CER1*, AT1G02205) and Ca^2+^-binding protein 1 (*ATCP1*, AT5G49480) for ABA-induced genes and *WALL-ASSOCIATED KINASE 2 *(*WAK2*, AT1G21270) for ABA-repressed genes.

Figure 2 summarizes the Q-PCR analysis. For 15 out of 17 genes, the Q-PCR results match the microarray data. The two exceptions are *LTI78/RD29A *and *PI-PLC*, for which ABA-upregulation is observed in guard cells but not in leaves from the microarray analysis, while the Q-PCR results show ABA-upregulation in both guard cells and leaves. This 94.1% (= 32/34 comparisons) agreement between the microarray analysis and the Q-PCR experiments is comparable or better than that observed in other microarray studies [39]. The discrepancy most probably stems from the different techniques employed to measure gene expression. Several studies have noted that microarray analysis is less sensitive than Q-PCR and can underestimate the true extent of transcript accumulation [40,41], so observing this result for a minority of the genes (2 out of 17) is not surprising.

### *cis*-acting regulatory elements in ABA-responsive genes

Several *cis*-regulatory elements responsible for mediating ABA-, drought-, and cold-induced gene expression have been identified by biochemical and molecular techniques [42]. We were interested to determine whether these known motifs would be enriched in our gene sets, which would support our identification of these genes as ABA-regulated. In addition, we were interested to assess whether ABA-regulated genes of guard cells and leaves would exhibit differential usage of these promoter motifs, or differential positioning of the motifs within the promoter region.

The G-box-containing ABA-responsive elements (ABREs) and coupling element 3 (CE3) are classical *cis*-acting elements in ABA-responsive gene expression [43-46]. In addition, the core motif of the dehydration-responsive element/C-repeat (DRE/CRT) is a *cis*-acting element found in many drought-, high-salt- and cold-responsive genes in Arabidopsis and rice, and provides the binding site for DRE-related transcription factors such as DRE-Binding proteins (DREBs) [46,47]. MYB and MYC binding motifs are also found in the promoters of drought-inducible genes. Proteins of the MYB and MYC families function in mediating drought- and ABA-regulated gene expression [33,48] and some members of these families such as AtMYC2 and AtMYB2 are synthesized following accumulation of endogenous ABA [49]. Other related *cis*-acting elements include the binding site of C-repeat factors (CBFs) and the low temperature responsive element (LTRE) [46].

We performed a statistical analysis (see Methods) of the promoter sequences (defined as the 1000-bp upstream regions) of ABA-responsive genes in guard cells and leaves, to check whether the known motifs would be enriched in our gene sets. The results of this analysis are shown in Table 1. We can see that ABREs are significantly enriched in guard cell ABA-induced genes. In particular, more than 50% of ABA-induced genes in guard cells have ABREs in their promoter regions, supporting the reliability of our microarray methods and predictions. DREs and LTREs are also highly enriched in guard cell ABA-induced genes. ABREs and the cold-related *cis*-acting element LTRE, but not DRE/CRT elements are also highly enriched in leaf ABA-induced genes. A motif over-represented in light-induced promoters, GCCAC (SORLIP1AT in the PLACE database [46]), also is found to be highly enriched in guard cell ABA-regulated genes but not in leaves. Compared with other motifs, CE3 has only a few matches in the ABA-regulated genes of either sample type. This is consistent with the findings that CE3 is well-represented in rice but almost absent in Arabidopsis [50]. None of the *cis*-acting elements enriched in ABA-induced genes is significantly enriched in ABA-repressed genes of guard cells or leaves, except the MYC binding site and LTRE. The mechanisms of ABA-repression are less well characterized than those for induction [33] and motifs specific for ABA-repressed genes may exist but have not been revealed yet (see next section).

**Table 1:** Enrichment of known *cis*-acting regulatory elements in 1000-bp region upstream of ABA-regulated genes

Motif name	Motif sequence	All genes	GC_up (696)		GC_down (477)		LF_up (596)		LF_down (441)	
		
		Hits	Hits	*P*-value	Hits	*P*-value	Hits	*P*-value	Hits	*P*-value
ABRE	(C/G/T)ACGTG(G/T)(A/C)	3178	382	**7.1E-149**	49	0.99	188	**2.3E-29**	57	0.71
CE3	ACGCGTGTC	24	7	**3.5E-7**	0	0.40	6	**1.9E-6**	1	0.08
DRE/CRT	(A/G)CCGAC	2748	137	**2.0E-9**	56	0.56	85	0.04	58	0.22
CBF binding	(A/G)(C/T)CGAC	6236	284	**3.6E-15**	131	0.47	215	**1.2E-6**	133	0.09
LTRE	CCGAC	5062	229	**2.0E-11**	106	0.48	162	**1.8E-3**	119	**7.7E-3**
MYB binding	(C/T)AAC(G/T)G	9108	313	**3.2E-3**	213	0.02	246	0.25	195	0.03
MYC binding	CACATG	4909	180	**2.7E-3**	138	**5.5E-5**	165	**1.5E-4**	118	**3.8E-3**
SORLIP1AT	GCCAC	6609	321	**1.4E-22**	152	0.08	189	0.07	136	0.19

'All genes' means all genes represented in the Affymetrix ATH1 chips. "GC_up" and "GC_down" represent ABA-induced and ABA-repressed genes in guard cells, respectively. "LF_up" and "LF_down" mean ABA-induced and ABA-repressed genes in leaves, respectively. The numbers in brackets in the header denote the total number of ABA-regulated genes in the corresponding category. *P*-values smaller than 0.01 are marked in bold.

**Figure 3:**
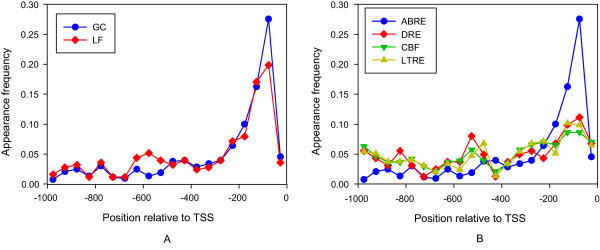
**Positional distribution of the *cis*-acting regulatory elements upstream of ABA regulated genes**. Data are summarized as data points with a bin size of 50 bases. TSS: transcription start site. (A) ABREs in guard cell and leaf ABA-induced genes, respectively. GC: guard cells, LF: leaves, (B) ABRE, DRE, CBF and LTBE in guard cell ABA-induced genes.

We also examined the positional preference of the *cis*-acting regulatory elements ABRE, DRE, CBF and LTRE in a 1000-bp region upstream of identified ABA-regulated genes of guard cells and leaves (Figure 3). There is no significant difference between ABRE locations in guard cell vs. leaf genes (Figure 3A); the same is true for the *cis*-acting elements DRE, CBF and LTRE locations in guard cell genes and leaf genes (not shown). The positional distribution of these four elements upstream of guard cell ABA-induced genes is shown in Figure 3B. ABREs are more likely than the other three motifs to be located in regions close to transcription start sites (TSS), with around 28% and 20% ABREs located in the -100~-50-bp region upstream of the TSS in guard cell and leaf ABA-induced genes, respectively.

### Identification of new promoter motifs

In guard cells, 39 of the ABA-induced genes have none of the known motifs of Table 1 in their promoter regions, and this is also true for 79 of the genes induced by ABA in leaves. In addition, not all genes containing a known ABA-related promoter motif show ABA-regulation in both tissue types. Accordingly, we sought to identify possible new motifs that may be involved in ABA-regulated gene expression, particularly of a cell-specific nature. We were also interested in the possibility of identifying repression-specific motifs. We identified all 5-10mer motifs highly enriched in the 1000-bp sequences upstream of ABA-regulated genes in guard cells and leaves (see Methods), and also compared their enrichment in previously published ABA-regulated gene sets. The statistically significant 5-mer motifs are summarized in Table 2. These motifs include not only the core of G-box-containing ABREs (ACGTG), subsequences of ABREs, the LTRE (CCGAC), and the light-induced motif (GCCAC), as expected from the above analysis, but also some new, previously unidentified, motifs. The results for 5-10mer motifs also show that there are many more significant motifs in ABA-induced genes than in ABA-repressed genes. In addition, a greater number of distinct motifs were found in guard cell ABA-induced genes than in leaf ABA-induced genes.

**Table 2:** 5-mer motifs significantly enriched in 1000-bp regions upstream of guard cell or leaf ABA-regulated genes

5-mer motifs	ABA-induced genes		5-mer motifs	ABA-repressed genes	
			
	GC (*P*< 10^-10^)	LF(*P *< 10^-10^)		GC (*P*< 10^-4^)^a^	LF(*P *< 10^-4^) ^a^
**ACGTG**	7.5E-90	4.2E-32	CCACT	4.3E-07	2.6E-04
**CACGT**	1.2E-84	1.3E-33	CCAAC	2.1E-06	9.6E-04
CCACG	5.7E-56	4.1E-11	CAACT	3.0E-05	1.4E-04
ACACG	1.5E-51	5.8E-21	CACAT	7.2E-05	1.1E-03
**CGTGT**	1.7E-47	1.1E-22	GGTCC	1.3E-04	7.7E-07
**CGTGG**	5.6E-36	2.0E-08	TGCAA	2.5E-03	1.1E-05
**GTGTC**	1.7E-24	2.5E-03	GTCCC	7.5E-04	2.8E-05
GCCAC	8.6E-23	>0.01	GACCA	>0.01	5.3E-05
**GTGGC**	1.3E-16	5.9E-05			
GACAC	5.9E-15	>0.01			
**GACGT**	7.3E-13	3.1E-07			
CACGC	1.2E-12	1.2E-05			
**TACGT**	5.5E-12	6.6E-12			
GTCGG	9.9E-12	6.8E-03			
CCGAC	1.5E-11	1.5E-03			
ACGTA	1.7E-11	1.3E-13			
TCCAC	2.9E-11	2.2E-05			
TGTCG	8.9E-11	5.8E-03			

Motifs marked in bold are subsequences of ABRE considered in Table 1. Underlined motifs exhibit tissue specificity (GC vs LF) based on our analysis (Additional file 3). For convenient comparison of GC and LF, *P*-values falling beyond the thresholds (see Methods) are also listed. ^a^Motifs appearing in more than 15000 loci are not listed.

To check whether newly identified 5-mer motifs significantly enriched in our gene sets are guard cell- or leaf-specific, we merged previously published ABA-regulated gene sets in Arabidopsis from [18-27] and compared the enrichment of these motifs. Note that none of these studies uses guard cells or rosette leaves as plant materials. We calculated the enrichment *P*-values of these motifs in the merged ABA-regulated gene set using all genes in the whole genome as the background, and also calculated the enrichment of these motifs in our gene sets using the merged ABA-regulated gene set as the background. The results are shown in Table S1 and Table S2 in Additional file 3, respectively. Guard cell-specific or leaf-specific motifs are designated as those that are not significantly enriched in the above merged ABA-regulated gene set, but are still significant in our guard cell or leaf ABA-regulated gene set when using the merged ABA-regulated gene set as the background. We find that GTCGG, which is not a subsequence of any known motifs, is significantly enriched in guard cell ABA-induced genes using either all genes in the whole genome or the merged ABA-regulated gene set as the background. By contrast, the enrichment of GTCGG in the merged ABA-regulated gene set is not significant (larger than 1.0E-03). We accordingly identify GTCGG as a potential guard cell-specific motif involved in ABA-induced gene expression. Similarly, TGCAA is a potential leaf-specific motif involved in ABA-repressed gene expression.

**Figure 4:**
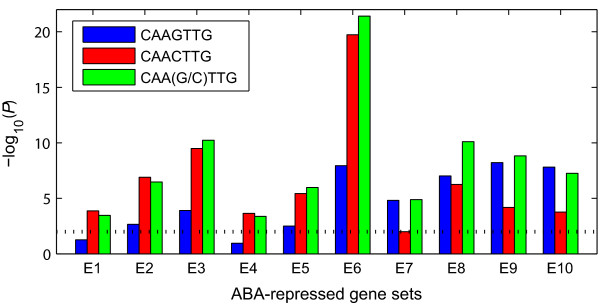
**Enrichment of the CAAGTTG motif, the CAACTTG motif and the CAA(G/C)TTG motif in 1000-bp upstream sequences of the ABA-repressed gene sets from ten transcriptomes**. E1: Hoth *et al.*, 2002 [20]; E2: Li *et al.*, 2006 [21]; E3: Xin *et al.*, 2005 [25]; E4: Matsui *et al.*, 2008 [19]; E5: Huang *et al.*, 2007 [26]; E6: Zeller *et al.*, 2009 [23]; E7: Sánchez *et al.*, 2004 [24]; E8: Nemhauser *et al.*, 2006 [22]; E9: this study (guard cells); E10: this study (leaves). The dotted line indicates the threshold for significance (*P *< 0.01)

Although only a few motifs emerge as significant from the promoters of ABA-repressed genes, in our analysis we find a novel strong candidate for a repression-specific motif, CAAGTTG, which is enriched in both guard cell ABA-repressed genes and leaf ABA-repressed genes (*P *= 6.0E-09 and 1.6E-08, respectively). This motif is very similar to the motif CAACTTG identified in [51], but in our guard cell and leaf gene sets, the enrichment of CAAGTTG is stronger than that of CAACTTG (*P *= 6.6E-05 and 1.7E-04, respectively). Known ABA-repressed genes *KAT2 *and *MYB60 *[14,16] contain the CAAGTTG motif. To examine whether this motif is also enriched in ABA-repressed genes in other tissues/cell types, we collected eight ABA-repressed gene sets from [19-26], and compared this motif's enrichment in these ABA-repressed gene sets (studies that report a very small number of ABA-repressed genes are not included here). As shown in Figure 4, CAAGTTG is highly enriched in the upstream sequences of most of the eight ABA-repressed gene sets, and the combined motif CAA(G/C)TTG is consistently enriched in all ABA-repressed gene sets, which further supports its involvement in ABA-mediated gene repression.

### Comparison with other ABA transcriptome studies

**Figure 5:**
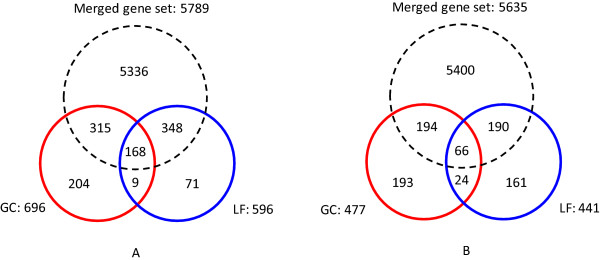
**Overlap of ABA-regulated genes in guard cells and leaves with previously published ABA-regulated gene sets**. (A) ABA-induced genes. (B) ABA-repressed genes. Studies contributing to the merged gene set were: Hoth *et al*., 2002 [20], Li *et al*., 2006 [21], Xin *et al*., 2005 [25], Matsui *et al*., 2008 [19], Huang *et al*., 2007 [26], Zeller *et al*., 2009 [23], Sánchez *et al*., 2004 [24], Nemhauser *et al*., 2006 [22], Leonhardt *et al*., 2004 [16], Seki *et al*., 2002 [18], Okamoto *et al*., 2010 [27].

Many transcriptome studies have been conducted on ABA regulation of gene expression in different Arabidopsis tissues and cell types, including whole plants [18,19], seedlings [20-26], seeds [27], and one previous study reporting ABA responsive genes in guard cells and mesophyll cells using a partial-genome (~8K) Affymetrix chip [16]. 5789 ABA-induced genes and 5635 ABA-repressed genes are obtained by merging the ABA-responsive genes in these studies. We examined how many of the ABA-regulated genes that we identified from our guard cell transcriptomes can be found in this merged ABA-regulated gene set. The overlap of our ABA-regulated genes with the previously published ABA-regulated genes is shown in Figure 5. We find that 69.4% of the ABA-induced genes and 54.5% ABA-repressed genes in guard cells identified in this work are found in previous ABA transcriptome studies. In leaves, 86.6% of ABA-induced genes and 58.0% ABA-repressed genes are found in previous ABA transcriptome studies. Such significant overlap supports the reliability of our microarray analysis.

**Figure 6:**
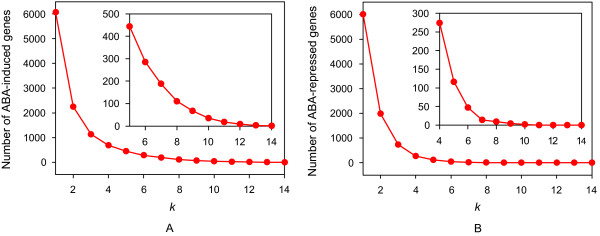
**Cumulative number of ABA-regulated genes that are common to different numbers of experiments**. (A) ABA-induced genes common to ≥ *k *experiments. (B) ABA-repressed genes common to ≥ *k *experiments. See Additional file 3 for a detailed description of the *k *experiments.

In addition to the overall comparison, we also conducted a pairwise comparison of the 11 ABA transcriptome studies mentioned above and our study, which describe a total of 14 different ABA-regulated transcriptomes (experiments) (Leonhardt *et al. *[16] contribute two experiments, and we contribute two as well). These transcriptome experiments have been done under various experimental conditions, including different ABA doses and treatment durations, tissue/cell types, microarray platforms, plant developmental stages, and methods for identifying ABA-regulated genes. A summary of experimental conditions for these ABA transcriptome studies is given in Table S3 (Additional file 3). The pairwise overlaps are measured by percentage (Additional file 3) and representation factor (see Methods), and their significance is examined by the hypergeometric distribution. The results are shown in Figure S1 (Additional file 3). Given the diversity of experimental parameters, it is not surprising that these studies do not have very high pairwise overlap percentages for ABA-regulated genes. However, when compared with random overlaps, all of them are significant for ABA-induced genes. For ABA-repressed gene sets, all have significant pairwise overlaps as compared with the expected overlap of two random sets except for the ABA-repressed gene sets in [16,18,27] which have non-significant overlaps with several other studies. Figure 6 shows the cumulative number of ABA-regulated genes that are common to different numbers of experiments. As expected from the pairwise overlap analysis, there are not many ABA-induced genes found in common as the number of experiments increases, and there are no ABA-repressed genes that are common to >11 experiments (transcriptomes). However, despite the diversity in tissue types, ABA treatments, and transcriptome platforms utilized in these experiments, we were able to find a "core set" of ~50 ABA-induced genes reported in any 9 out of 14 experiments (Figure 6A), and a similar number of "core set" ABA-repressed genes reported in any 6 out of 14 experiments (Figure 6B). Table 3 lists the core set of ABA-induced genes, and Table 4 lists the core set of ABA-repressed genes.

**Table 3:** 67 ABA-induced genes common to ≥ 9 experiments

Genes	Descriptions	No.
AT1G01470*^	LEA14 (LATE EMBRYOGENESIS ABUNDANT 14)	14
AT2G33380*^	RD20 (RESPONSIVE TO DESSICATION 20); calcium ion binding	13
AT3G11410*^	AHG3/ATPP2CA (ARABIDOPSIS THALIANA PROTEIN PHOSPHATASE 2CA)	13
AT1G20440*^	COR47 (cold regulated 47)	12
AT1G52690*^	late embryogenesis abundant protein, putative	12
AT1G72770*^	HAB1 (HOMOLOGY TO ABI1); protein serine/threonine phosphatase	12
AT2G41190*^	amino acid transporter family protein	12
AT2G47770*	benzodiazepine receptor-related	12
AT5G06760*^	late embryogenesis abundant group 1 domain-containing protein	12
AT1G08920*	sugar transporter, putative	11
AT1G77450*	ANAC032 (Arabidopsis NAC domain containing protein 32)	11
AT2G15970*^	COR413-PM1 (cold regulated 413 plasma membrane 1)	11
AT2G46270*^	GBF3 (G-BOX BINDING FACTOR 3); transcription factor	11
AT2G46680 ^	ATHB-7 (ARABIDOPSIS THALIANA HOMEOBOX 7); transcription factor	11
AT4G27410*	RD26 (RESPONSIVE TO DESSICATION 26); transcription factor	11
AT5G52310*^	COR78 (COLD REGULATED 78)	11
AT5G59320*	LTP3 (LIPID TRANSFER PROTEIN 3); lipid binding	11
AT5G66400*^	RAB18 (RESPONSIVE TO ABA 18)	11
AT1G04220*^	KCS2 (3-KETOACYL-COA SYNTHASE 2); fatty acid elongase	10
AT1G05100*^	MAPKKK18 (Mitogen-activated protein kinase kinase kinase 18)	10
AT1G16850*	unknown protein	10
AT1G20450	ERD10/LTI45 (EARLY RESPONSIVE TO DEHYDRATION 10)	10
AT1G49450*	transducin family protein/WD-40 repeat family protein	10
AT1G51140*	basic helix-loop-helix (bHLH) family protein	10
AT1G58270*	ZW9	10
AT3G11420	fringe-related protein	10
AT3G29575*	AFP3 (ABI FIVE BINDING PROTEIN 3)	10
AT3G61890 ^	ATHB-12 (ARABIDOPSIS THALIANA HOMEOBOX PROTEIN 12)	10
AT4G05100*	AtMYB74 (myb domain protein 74); DNA binding/transcription factor	10
AT4G24130*^	unknown protein	10
AT4G26080*	ABI1 (ABA INSENSITIVE 1); calcium ion binding	10
AT4G30960*	CIPK6 (CBL-INTERACTING PROTEIN KINASE 6); kinase	10
AT5G01520*	zinc finger (C3HC4-type RING finger) family protein	10
AT5G15960	Stress-induced protein KIN1	10
AT5G59220*	protein phosphatase 2C, putative/PP2C, putative	10
AT1G07430*	protein phosphatase 2C, putative	9
AT1G07720	KCS3 (3-KETOACYL-COA SYNTHASE 3)	9
AT1G21790*	similar to unnamed protein product [Vitis vinifera] (GB:CAO61872.1)	9
AT1G60190*	armadillo/beta-catenin repeat family protein/U-box domain-containing protein	9
AT1G62570*	FMO GS-OX4 (FLAVIN-MONOOXYGENASE GLUCOSINOLATE S-OXYGENASE 4)	9
AT1G62710*	BETA-VPE (vacuolar processing enzyme beta); cysteine-type endopeptidase	9
AT1G73480*	hydrolase, alpha/beta fold family protein	9
AT1G77120*	ADH1 (ALCOHOL DEHYDROGENASE 1)	9
AT2G17840 ^	ERD7 (EARLY-RESPONSIVE TO DEHYDRATION 7)	9
AT2G30360*	CIPK11 (SOS3-INTERACTING PROTEIN 4); kinase	9
AT2G30550*	lipase class 3 family protein	9
AT2G37870	protease inhibitor/seed storage/lipid transfer protein (LTP) family protein	9
AT2G39050*	hydroxyproline-rich glycoprotein family protein	9
AT2G47780*	rubber elongation factor (REF) protein-related	9
AT3G02480*	ABA-responsive protein-related	9
AT3G17520*^	late embryogenesis abundant domain-containing protein	9
AT3G22600*	protease inhibitor/seed storage/lipid transfer protein (LTP) family protein	9
AT3G48510	unknown protein	9
AT3G50970 ^	LTI30 (LOW TEMPERATURE-INDUCED 30)	9
AT3G55500*	ATEXPA16 (ARABIDOPSIS THALIANA EXPANSIN A16)	9
AT3G57010*	strictosidine synthase family protein	9
AT4G17550*	transporter-related	9
AT4G21440*^	ATM4/ATMYB102 (ARABIDOPSIS MYB-LIKE 102); transcription factor	9
AT4G23050*	protein kinase, putative	9
AT4G30470*	cinnamoyl-CoA reductase-related	9
AT4G33550	lipid binding	9
AT4G34000*^	ABF3/DPBF5 (ABSCISIC ACID RESPONSIVE ELEMENTS-BINDING FACTOR 3)	9
AT5G11110*	ATSPS2F/SPS1 (SUCROSE PHOSPHATE SYNTHASE 1)	9
AT5G52300*^	LTI65/RD29B (RESPONSIVE TO DESSICATION 29B)	9
AT5G57050*	ABI2 (ABA INSENSITIVE 2); protein serine/threonine phosphatase	9
AT5G59310*	LTP4 (LIPID TRANSFER PROTEIN 4); lipid binding	9
AT5G61820*	similar to MtN19-like protein [Pisum sativum] (GB:AAU14999.2)	9

The first column lists the AGI locus identifiers of the genes. *Genes that appear in our guard cell ABA-induced gene set. ^Genes that appear in Leonhardt et al.'s guard cell ABA-induced gene set [16]. The genes that appear in our ABA-induced gene set are marked with underlines if they are not represented on the Affymetrix ~8K chips used in [16]. The second column gives brief TAIR descriptions for the genes. The third column indicates the number of experiments that the genes are found common to.

**Table 4:** 47 ABA-repressed genes common to ≥ 6 experiments

Genes	Descriptions	No.
AT2G38310*	unknown protein	10
AT4G21870*	26.5 kDa class P-related heat shock protein (HSP26.5-P)	10
AT1G66940*	protein kinase-related	9
AT1G69530*	ATEXPA1 (ARABIDOPSIS THALIANA EXPANSIN A1)	9
AT1G03870*	FLA9	8
AT1G08930	ERD6 (EARLY RESPONSE TO DEHYDRATION 6)	8
AT4G36670*	mannitol transporter, putative	8
AT5G02760*	protein phosphatase 2C family protein	8
AT5G05440*	unknown protein	8
AT1G14210*	ribonuclease T2 family protein	7
AT3G01860*	unknown protein	7
AT3G14840*	leucine-rich repeat family protein/protein kinase family protein	7
AT3G50740	UGT72E1 (UDP-glucosyl transferase 72E1)	7
AT4G17460*	HAT1 (homeobox-leucine zipper protein 1); DNA binding/transcription factor	7
AT1G07090	LSH6 (LIGHT SENSITIVE HYPOCOTYLS 6)	6
AT1G08810*^	MYB60 (myb domain protein 60); DNA binding/transcription factor	6
AT1G29430	auxin-responsive family protein	6
AT1G51850	leucine-rich repeat protein kinase, putative	6
AT1G52190	proton-dependent oligopeptide transport (POT) family protein	6
AT1G56430	NAS4 (NICOTIANAMINE SYNTHASE 4); nicotianamine synthase	6
AT1G68840	RAV2 (REGULATOR OF THE ATPASE OF THE VACUOLAR MEMBRANE)	6
AT2G18300	basic helix-loop-helix (bHLH) family protein	6
AT2G23600 ^	ACL (ACETONE-CYANOHYDRIN LYASE); hydrolase	6
AT2G40330	Bet v I allergen family protein	6
AT2G46450	ATCNGC12 (cyclic nucleotide gated channel 12); cyclic nucleotide binding	6
AT3G14310*^	ATPME3 (Arabidopsis thaliana pectin methylesterase 3)	6
AT3G23880*	F-box family protein	6
AT3G49260*	IQD21 (IQ-DOMAIN 21, IQ-domain 21); calmodulin binding	6
AT3G49670	BAM2 (big apical meristem 2); ATP binding/protein serine/threonine kinase	6
AT3G49940*	LBD38 (LOB DOMAIN-CONTAINING PROTEIN 38)	6
AT4G15390	transferase family protein	6
AT4G17870*	similar to unknown protein [Arabidopsis thaliana] (TAIR:AT4G17870.1)	6
AT4G21410*	protein kinase family protein	6
AT4G24780	pectate lyase family protein	6
AT4G36540	BEE2 (BR ENHANCED EXPRESSION 2); DNA binding/transcription factor	6
AT4G38840	auxin-responsive protein, putative	6
AT5G07580*	DNA binding/transcription factor	6
AT5G14120*	nodulin family protein	6
AT5G14760*	AO (L-ASPARTATE OXIDASE); L-aspartate oxidase	6
AT5G25460	unknown protein	6
AT5G25840*	unknown protein	6
AT5G39080*	transferase family protein	6
AT5G61590	AP2 domain-containing transcription factor family protein	6
AT5G63180	pectate lyase family protein	6
AT5G64100	peroxidase, putative	6
AT5G66690	UGT72E2; UDP-glycosyltransferase/coniferyl-alcohol glucosyltransferase	6
AT5G66770	scarecrow transcription factor family protein	6

The first column lists the AGI locus identifiers of the genes. *Genes that appear in our guard cell ABA-repressed gene set. ^Genes that appear in Leonhardt et al.'s guard cell ABA-repressed gene set [16]. The genes that appear in our ABA-repressed gene set are marked with underlines if they are not represented on the Affymetrix ~8K chips used in [16]. The second column gives brief TAIR descriptions for the genes. The third column indicates the number of experiments that the genes are found common to.

### Identification of new guard cell ABA-responsive genes

In addition to identifying core sets of ABA-induced and ABA-repressed genes, we also discovered a number of novel ABA-responsive genes in both guard cells and leaves. In particular, our results provide a valuable comprehensive source of ABA-regulated genes in guard cells since the smaller Affymetrix ~8K array was used in [16] and therefore could not provide information on ~2/3 of the transcriptome. For example, among 1173 genes significantly ABA-regulated in our guard cell transcriptome, 1122 genes (658 ABA-induced and 464 ABA-repressed) were not previously reported to be ABA-regulated by Leonhardt *et al. *[16]. Of these 1173 genes, 148 ABA-induced genes and 149 ABA-repressed genes were also not reported as ABA-regulated in any of the 11 previous transcriptome studies we analyzed (see Figure 5 legend for a list of these studies). The proposed guard cell-specific ABA-induction motif, GTCGG, is significantly enriched (*P *< 1.3E-04) in the 148 newly identified ABA-induced genes of guard cells, consistent with the hypothesis that this motif participates in ABA-regulation of gene expression in guard cells. We also identified a smaller set of ABA-regulated transcripts in leaves that have not been reported in the previous ABA microarray studies: 62 ABA-induced genes and 113 ABA-repressed genes. In Table 5 and Table 6 we list by *P*-value the top 50 new ABA-induced genes and ABA-repressed genes in guard cells not previously reported by any of the 11 previous ABA microarray studies that we evaluated. Additional file 4 and Additional file 5 list all the new ABA-responsive genes in guard cells and leaves, respectively.

**Table 5:** Top 50 ABA-induced genes in guard cells that have not been reported in other ABA transcriptome studies

AGI #	Gene name	Description	*P*-value	Fold change
AT4G12130		aminomethyltransferase	1.2E-12	6.1
AT3G01510		5'-AMP-activated protein kinase beta-1 subunit-related	2.5E-12	8.5
AT3G15357		unknown protein	2.7E-12	31.2
AT5G13930	ATCHS	CHALCONE SYNTHASE	4.7E-11	261.3
AT3G04460	ATPEX12	PEROXIN-12; actin binding	6.3E-11	3.7
AT2G19580	TET2	TETRASPANIN2	1.1E-10	4.3
AT2G43018		conserved peptide upstream open reading frame 17	5.7E-10	4.4
AT3G14180		transcription factor	1.2E-09	3.6
AT1G32550		ferredoxin family protein	1.3E-09	4.9
AT5G17980		C2 domain-containing protein	1.5E-09	5.0
AT5G67370		unknown protein	1.8E-09	4.5
AT2G44660		transferase, transferring glycosyl groups	1.8E-09	3.9
AT3G18170		similar to unknown protein AT3G18180.1	1.9E-09	3.3
AT4G08980		F-box family protein (FBW2)	2.4E-09	3.6
AT1G68470		exostosin family protein	2.4E-09	10.3
AT2G24150	HHP3	heptahelical protein 3; receptor	2.6E-09	10.0
AT5G60790	ATGCN1	Arabidopsis thaliana general control non-repressible 1	4.3E-09	4.8
AT5G61670		heat shock protein binding/unfolded protein binding	5.0E-09	3.0
AT3G23580	RNR2	RIBONUCLEOTIDE REDUCTASE 2A	6.0E-09	6.1
AT4G17420		similar to unknown protein AT5G47420.1	7.6E-09	3.8
AT1G67960		similar to unnamed protein product GB:CAO42391.1	8.2E-09	2.2
AT5G45920		carboxylesterase	1.3E-08	5.9
AT5G16990		NADP-dependent oxidoreductase, putative	1.5E-08	2.8
AT2G35700	ERF38	ERF FAMILY PROTEIN 38; transcription factor	1.6E-08	2.5
AT4G11370	RHA1A	RING-H2 finger A1A; zinc ion binding	1.7E-08	5.9
AT2G04400		indole-3-glycerol phosphate synthase (IGPS)	1.8E-08	3.7
AT5G07250	ATRBL3	ARABIDOPSIS RHOMBOID-LIKE PROTEIN 3	2.3E-08	3.4
AT3G13040		myb family transcription factor	2.4E-08	2.8
AT4G00370	ANTR2	anion transporter 2	2.5E-08	2.7
AT4G34930		1-phosphatidylinositol phosphodiesterase-related	2.7E-08	3.4
AT1G01570		fringe-related protein	2.8E-08	2.7
AT1G23750		DNA-binding protein-related	2.9E-09	3.1
AT5G63330		DNA-binding bromodomain-containing protein	3.4E-08	3.1
AT1G18360		hydrolase, alpha/beta fold family protein	3.7E-08	6.2
AT5G55090	MAPKKK15	Mitogen-activated protein kinase kinase kinase 15	4.8E-08	5.9
AT1G63710	CYP86A7	cytochrome P450, family 86, subfamily A, polypeptide 7	5.9E-08	10.6
AT2G29730	UGT71D1	UDP-GLUCOSYL TRANSFERASE 71D1	7.5E-08	8.1
AT5G03160	ATP58IPK	ARABIDOPSIS HOMOLOG OF MAMALLIAN P58IPK	8.8E-08	2.6
AT3G50850		similar to unknown protein AT5G49560.1	1.0E-07	3.7
AT1G03630	POR C	PROTOCHLOROPHYLLIDE OXIDOREDUCTASE	1.1E-07	2.8
AT2G39020		GCN5-related N-acetyltransferase (GNAT) family	1.2E-07	3.6
AT1G10960	ATFD1	FERREDOXIN 1; 2 iron, 2 sulfur cluster binding	1.2E-07	2.5
AT1G19190		hydrolase	1.8E-07	6.7
AT2G45300		3-phosphoshikimate 1-carboxyvinyltransferase	1.8E-07	2.9
AT2G44060		late embryogenesis abundant family protein	1.9E-07	3.3
AT4G10730		kinase	2.1E-07	2.9
AT2G47670		invertase/pectin methylesterase inhibitor family protein	2.1E-07	3.1
AT3G54960	ATPDIL1-3	PDI-LIKE 1-3; thiol-disulfide exchange intermediate	2.3E-07	3.6
AT4G22320		similar to unknown protein TAIR:AT5G55210.1	2.6E-07	1.7
AT5G61370		pentatricopeptide (PPR) repeat-containing protein	2.6E-07	5.2

The fold changes are calculated by averaging three biological replicates and comparing the average expression values with ABA treatment to those without ABA treatment. The genes with underlines also appear in our leaf ABA-induced genes.

**Table 6:** Top 50 ABA-repressed genes in guard cells that have not been reported in other ABA transcriptome studies

AGI #	Gene name	Description	*P*-value	Fold change
AT2G45120		zinc finger (C2H2 type) family protein	4.7E-15	14.7
AT1G60630		leucine-rich repeat family protein	8.2E-13	6.7
AT3G19850		phototropic-responsive NPH3 family protein	3.3E-12	13.3
AT1G19620		unknown protein	1.1E-11	9.0
AT1G11340		S-locus lectin protein kinase family protein	1.5E-11	23.6
AT4G04955	ATALN	ARABIDOPSIS ALLANTOINASE	2.8E-11	3.3
AT3G19120		unknown protein	3.4E-11	10.6
AT4G37870	PCK1	PHOSPHOENOLPYRUVATE CARBOXYKINASE 1	3.7E-11	4.5
AT4G01770	RGXT1	RHAMNOGALACTURONAN XYLOSYLTRANSFERASE 1	1.2E-10	7.3
AT5G47780	GAUT4	Galacturonosyltransferase 4	2.4E-10	4.2
AT5G52120	ATPP2-A14	Phloem protein 2-A14; carbohydrate binding	2.9E-10	6.1
AT1G21540		AMP-binding protein, putative	4.6E-10	6.8
AT1G09010		glycoside hydrolase family 2 protein	7.3E-10	3.4
AT1G28010	PGP14	P-GLYCOPROTEIN 14; ATPase	1.2E-09	12.6
AT3G52870		calmodulin-binding family protein	1.7E-09	10.2
AT1G75880		family II extracellular lipase 1 (EXL1)	2.5E-09	4.2
AT2G25780		unknown protein	3.8E-09	48.1
AT2G45810		DEAD/DEAH box helicase, putative	6.7E-09	4.1
AT3G62780		C2 domain-containing protein	7.6E-09	6.7
AT5G57580		calmodulin-binding protein	1.5E-08	2.8
AT3G09770		zinc finger (C3HC4-type RING finger) family protein	1.6E-08	3.3
AT4G08920	CRY1	CRYPTOCHROME 1	1.7E-08	4.8
AT2G34160		nucleic acid binding	1.7E-08	4.9
AT3G24140	FMA	FMA (FAMA); DNA binding/transcription activator	1.9E-08	5.3
AT1G75450	CKX5	CYTOKININ OXIDASE 5; cytokinin dehydrogenase	2.1E-08	2.9
AT3G11540	SPY	SPINDLY; transferase, transferring glycosyl groups	2.1E-08	2.6
AT3G51390		zinc finger (DHHC type) family protein	2.3E-08	2.8
AT1G12730		cell division cycle protein-related	2.5E-08	5.3
AT1G11260	STP1	SUGAR TRANSPORTER 1	2.6E-08	3.3
AT2G16640	ATTOC132	MULTIMERIC TRANSLOCON COMPLEX IN THE OUTER ENVELOPE MEMBRANE 132	2.9E-08	2.7
AT5G53890		leucine-rich repeat transmembrane protein kinase	3.3E-08	3.3
AT5G11580		UVB-resistance protein-related	3.6E-08	2.7
AT2G22300	SR1	SIGNAL RESPONSIVE 1; calmodulin binding	4.5E-08	6.5
AT5G23880	CPSF100	CLEAVAGE AND POLYADENYLATION SPECIFICITY FACTOR	4.6E-08	1.8
AT2G44065		ribosomal protein L2 family protein	5.1E-08	2.9
AT3G17020		universal stress protein (USP) family protein	5.2E-08	2.7
AT1G74040	IMS1	IMS1; 2-isopropylmalate synthase	5.4E-08	3.7
AT1G17230		protein binding/protein kinase	5.5E-08	3.4
AT3G05160		sugar transporter, putative	5.9E-08	4.8
AT2G37420		kinesin motor protein-related	6.0E-08	11.0
AT1G63120	ATRBL2	ARABIDOPSIS THALIANA RHOMBOID-LIKE 2	6.3E-08	3.1
AT5G64560		magnesium transporter CorA-like family protein	8.8E-08	2.4
AT5G63620		zinc-binding dehydrogenase family protein	9.3E-08	2.6
AT4G26830		hydrolase, hydrolyzing O-glycosyl compounds	9.9E-08	7.3
AT1G21400		2-oxoisovalerate dehydrogenase, putative	1.3E-07	3.0
AT5G48950		thioesterase family protein	1.3E-07	5.2
AT5G37450		leucine-rich repeat transmembrane protein kinase	1.4E-07	9.9
AT3G05660	AtRLP33	Receptor Like Protein 33; kinase/protein binding	1.5E-07	3.1
AT3G58840		similar to myosin heavy chain-related AT1G06530.1	1.8E-07	3.9
AT4G38300		glycosyl hydrolase family 10 protein	2.1E-07	3.8

The fold changes are calculated by averaging three biological replicates and comparing the average expression values with ABA treatment to those without ABA treatment. The genes with underlines also appear in our leaf ABA-repressed genes.

### Functional enrichment in ABA-responsive genes

**Figure 7:**
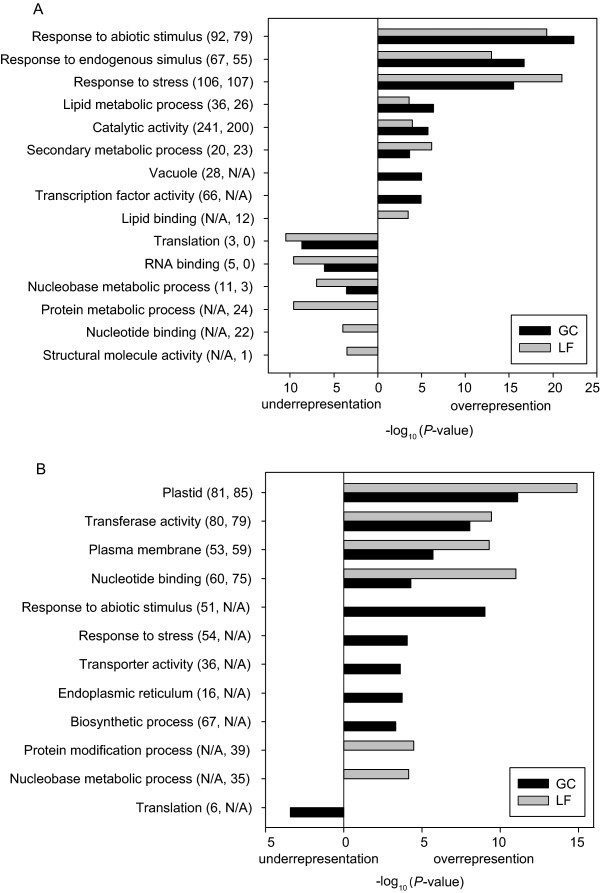
**Significantly enriched functional categories in guard cell and leaf ABA-responsive genes**. (A) ABA-induced genes. (B) ABA-repressed genes. Numbers in parentheses indicate the numbers of genes annotated with the corresponding functional categories in guard cell and leaf ABA-regulated gene sets respectively. 'N/A' means the enrichment of the function is not significant.

ABA is involved in a myriad of biological processes, including plant growth and development, and plant responses to environmental stresses such as cold, drought, and pathogens. To examine whether ABA-responsive genes identified in our study tend to be involved in such aspects of plant physiology, we used the GO functional analysis tool BiNGO 2.42 embedded in the Cytoscape project [52,53] to functionally categorize our ABA-responsive genes in guard cells and leaves. TAIR9 GO data (GOSlim_Plants) [54] for the whole Arabidopsis gene annotation were used as the reference set. The significance of overrepresentation and underrepresentation of a functional category was calculated by the hypergeometric cumulative distribution. Bonferroni familywise error rate (FWER) correction was applied for multiple testing. The functional categories with *P*-values less than 1.0E-03 that also have FWER -corrected *P*-values smaller than 0.05 are given in Figure 7.

For the ABA-induced genes, the three most significant functional categories in both guard cells and leaves are *response to abiotic stimulus*, *response to endogenous stimulus*, and *response to stress *(Figure 7A), all consistent with the fact that ABA plays important roles in the regulation of gene expression during stress responses. *Lipid metabolic process*, *catalytic activity *and *secondary metabolic process *are also enriched in both guard cell and leaf ABA-induced genes but the enrichment of *vacuole *and *transcription factor activity *is unique to guard cells. In addition, several functional categories are underrepresented within ABA-induced genes, including *translation*, *RNA binding*, *protein metabolism*, and *nucleobase metabolism *(see Discussion).

ABA-repressed genes show enrichment of different functional categories than ABA-induced genes (Figure 7B). The functional categories enriched in both guard cell and leaf ABA-repressed genes are *plastid*, *transferase activity*, *plasma membrane*, and *nucleotide binding*. *Response to abiotic stimulus, response to stress *and several other functional categories are highly enriched in guard cell ABA-repressed genes but not in leaf ABA-repressed genes. Two functional categories, *nucleobase metabolic process *and *protein modification process*, are enriched in leaf ABA-repressed genes but not in guard cell ABA-repressed genes. The functional categories *protein metabolism*, *nucleobase metabolism *and *nucleotide binding *which are underrepresented in leaf ABA-induced genes are overrepresented in leaf ABA-repressed genes.

### Guard cell signaling and development genes regulated at the transcript level by ABA

**Figure 8:**
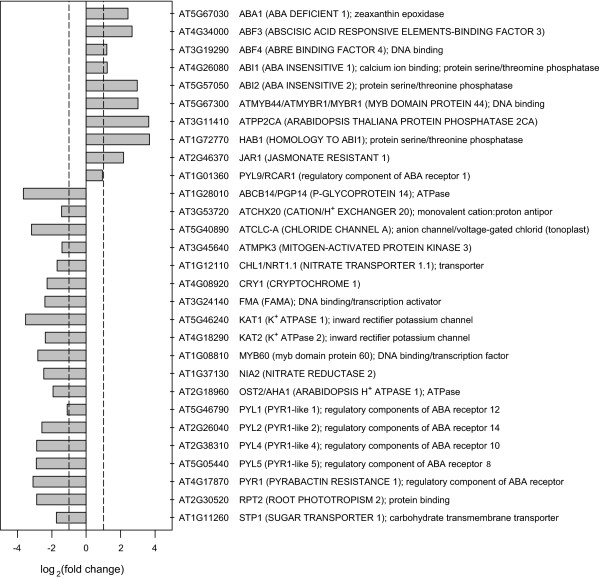
**Known guard cell signaling and development genes regulated by ABA at the transcript level**. The *x *axis denotes the log2 ratio of average gene expression with ABA treatment over that without ABA treatment. The *y *axis lists known guard cell signaling and development genes. The dashed lines indicate 2-fold change. All genes depicted showed significant regulation of gene expression based on FDR-corrected *P*-value of the linear model method, and also passed the correlation threshold of the Boolean method.

A number of genes and proteins in guard cells have been revealed to function in stomatal movements in response to stimuli, including ABA, CO_2_, light and pathogens. In [55], a total of 67 guard cell signaling genes was compiled from published literature. Li and Assmann [56] reviewed selected guard cell signaling and development genes. In addition, Kwak *et al. *collected 69 guard cell signaling genes [13]. We combined these sets as well as 22 additional genes with functional guard cell roles as described by Kim *et al. *[14] and 24 genes involved in guard cell patterning and development as described by Dong and Bergmann [57], to obtain a list of 149 known guard cell signaling or development genes/proteins (Additional file 6). Of these guard cell components, 29 are significantly regulated by ABA at the transcript level (Figure 8).

### Cross-regulation by ABA and heterotrimeric G proteins

**Figure 9:**
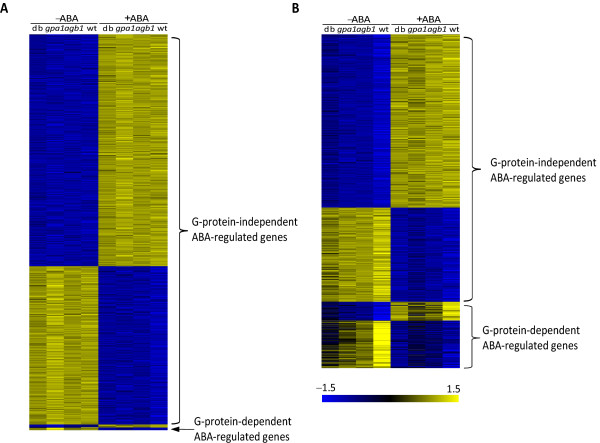
**Expression patterns of ABA-responsive genes in guard cells and leaves**. (A) Expression patterns of ABA-regulated genes in guard cells. (B) Expression patterns of ABA-regulated genes in leaves. The rows of each pattern correspond to genes, and the columns correspond to conditions, i.e. four genotypes, *agb1 gpa1 *double mutant (db), *gpa1 *mutant, *agb1 *mutant, wild-type (wt), without or with ABA treatment.

Heterotrimeric G-proteins, composed of α, β and γ subunits, participate in a wide range of crucial signaling pathways in eukaryotes [58,59]. ABA signaling is known to interact with heterotrimeric G-protein signaling in both developmental and stress responses [60-63]. In a previous study, we found that ABA signaling also cross-talks with G protein signaling at the level of the transcriptome [31]. In the present study, we used the genome-wide guard cell microarray data from wild-type, Gα (*gpa1*), Gβ (*agb1*) and Gγ double (*agb1 gpa1*) mutant plants, with and without ABA treatment to systematically investigate ABA regulation of gene expression in guard cells and compared it with the ABA-regulated transcriptome of leaves from the same genotypes. We identified a number of G-protein-independent ABA-regulated genes and G-protein-dependent ABA-regulated genes, which will facilitate the screening and identification of novel guard cell signaling genes. Figure 9 demonstrates the heat map of expression patterns of these genes (drawn by Matrix2png [64]) and shows that in both guard cells and leaves the number of G-protein-independent ABA-regulated genes is dominant, consistent with the fact that ABA mediates many stress and developmental signaling pathways without any known participation from the heterotrimeric G protein. The number of G-protein-dependent ABA-regulated genes in leaves is much larger than in guard cells, suggesting that the G-protein may be more involved in ABA signaling in mesophyll cells than in guard cells. Six (out of 9) guard cell G-protein-dependent ABA-induced genes and 14 (out of 58) leaf G-protein-dependent ABA-induced genes have ABREs in their promoter regions, which is statistically significant.

### Cross-regulation by ABA and other hormones

The phenomenon of cross-talk between plant hormones is well-established at the physiological level [10,65,66]. Nemhauser et al. [22] performed an analysis of seven hormone-treated transcriptomes generated by Goda *et al. *[67] from 7-day-old Arabidopsis seedlings and identified target genes regulated by the hormones abscisic acid (ABA), gibberellic acid 3 (GA), indole-3-acetic acid (IAA, auxin), 1-amino-cyclopropane-1-carboxylic acid (ACC, ethylene precursor), zeatin (CK, cytokinin), brassinolide (BL, brassinosteroid), and methyl jasmonate (MJ, jasmonate). Six of these seven hormones both positively and negatively regulate significant numbers of genes at the transcript level, identified by stringent analysis using two different methods [22], whereas the seventh hormone, GA, regulates only a few genes identified by a low-stringency linear model [22]. We took the intersection of the hormone-regulated gene sets identified by the rank product and linear model methods used in [22] and compared our ABA-regulated genes in guard cells and leaves with these hormonally regulated genes. The overlap of our ABA-regulated genes with hormone-regulated gene sets was calculated both in the same direction, that is, genes up- (down-) regulated by a hormone are compared with genes up- (down-) regulated by another hormone, and in the antagonistic direction. Representation factor and *P*-values calculated by the hypergeometric distribution (see Methods) were used to evaluate the overlaps.

**Figure 10:**
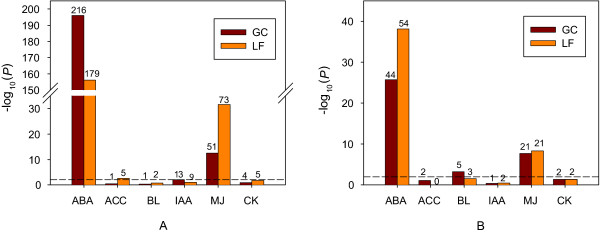
**Comparison of our ABA-responsive genes with the hormone-regulated genes in **[22]. GC: guard cell; LF: leaf. (A) Hormone-induced genes. The numbers of hormone-induced genes in [22] are: ABA 512, ACC 34, BL 28, IAA 198, MJ 522, and CK 60. (B) Hormone-repressed genes. The numbers of hormone-repressed genes in [22] are: ABA 270, ACC 23, BL 33, IAA 61, MJ 231, and CK 16. The dashed lines indicate the threshold for significance (*P *< 0.01). The numbers over the bars represent the numbers of our ABA-regulated genes that overlap with the hormone-regulated genes in [22].

Figure 10 shows the significance of the overlaps of our ABA-regulated genes in guard cells and leaves with the six hormone-regulated gene sets of Nemhauser *et al. *[22]. Our ABA-regulated genes in both guard cells and leaves have highly significant overlap with ABA-regulated and MJ-regulated genes in [22]. ABA-induced genes have a larger overlap with MJ-induced genes in leaves than in guard cells. Although the numbers are smaller, guard cell ABA-induced genes also have a significant overlap with IAA-induced genes, and leaf ABA-induced genes have a significant overlap with ACC-induced genes. Guard cell ABA-repressed genes have a significant overlap with BL-repressed genes. Overlap of our ABA-regulated genes with hormone-regulated gene sets in an antagonistic way, although not significant, is observed in each hormone-regulated gene set (Table S4 in Additional file 3). In some instances, the extent of antagonistic overlap is similar to or even larger than that corresponding to the comparison in the same direction. For example, ABA-repressed genes in guard cells and leaves have 10 and 8 genes in common with IAA-induced genes, and ABA-induced genes in guard cells and leaves have 14 and 13 common genes with MJ-repressed genes. Nemhauser *et al. *defined marker genes as those genes specifically regulated by one hormone in a high stringency analysis and not by any other hormone even in a lower stringency analysis [22]. However, some genes designated as marker genes specific for hormones other than ABA are actually found to be ABA-regulated in our guard cell or leaf transcriptomes (Table S5 and Table S6 in Additional file 3).

## Discussion

Our identified ABA-regulated guard cell and leaf transcriptomes are supported not only by Q-PCR verification but also by their significant overlap with the ABA-regulated transcriptomes of other tissues with respect to the promoter motifs that are utilized, the genes that are regulated, and the functional roles of the encoded gene products in stress tolerance as ascertained from GO analysis. However, as discussed in detail below, a deeper study of these new transcriptomes reveals many interesting features.

### Promoters

As expected, known promoter motifs conferring ABA- and stress-related gene regulation, such as ABREs and LTREs, are over-represented in the promoters of both our guard cell and leaf ABA-induced gene sets. However, promoters of ABA-regulated genes of guard cells but not leaves are enriched in MYB and DRE/CRT elements. These differences suggest differential utilization of specific transcription factors in guard cells vs. leaves. Functional support for this hypothesis is offered by published analyses of MYB family members and stomatal regulation. Two members of the Arabidopsis R2R3-MYB family, MYB15 and AtMYB44, are expressed in guard cells and their overexpression lines show enhancement of ABA-induced stomatal closure and drought tolerance [68,69]. Comparable functional data detailing guard cell roles for the DRE/CRT binding factors are presently lacking; our analysis of promoter motifs suggests that DRE binding proteins will also be found to participate in guard cell ABA responses. Analyses in other tissues have shown that genes with DRE elements in their promoters are regulated by dehydration but not by ABA, although ABA-sensitivity may be conferred by the concerted action of the DREB and ABF proteins [70]. When our guard cell ABA-regulated gene set is assessed for its complement of known transcription factors, we find that *DREB1A*, *ABF3 *and *ABF4 *show concerted ABA-induction in guard cells.

We hypothesized that, given the specialized nature of guard cell responses to ABA, these cells might also utilize specialized promoter motifs. One motif, GTCGG, which is not a subsequence of any known motifs, was thereby provisionally identified, awaiting functional verification. In addition, a greater number of different enriched motifs are found for guard cell ABA-induced genes than for leaf ABA-induced genes, suggesting that transcription factors in guard cells may target a greater diversity of *cis*-acting regulatory elements in ABA responses as compared to those in leaves.

Only a few motifs, including CAAGTTG, emerged as significant from the promoters of ABA-repressed genes. For both guard cells and leaves, there are more significant 5-10mer motifs in ABA-induced genes than in ABA-repressed genes. Since the numbers of ABA-induced and ABA-repressed genes are of similar magnitude in our datasets, this result suggests that evolution may have favored a greater diversity in mechanisms for regulation of gene repression than for gene induction, thus is it more difficult for any one ABA-repression motif to achieve statistical significance.

### Comparison with other ABA transcriptome studies

The fact that many classic ABA- and stress-related promoter motifs are enriched upstream of genes in our ABA-regulated gene sets led us to predict that many of the genes identified by our analyses would also be present in comparable transcriptomes derived from other tissues. Indeed, we find over half of all ABA-regulated genes in our guard cell and leaf datasets were also identified in other studies, with the highest percentage overlap (86.6%) found for ABA-induced genes in our leaf gene set, probably reflecting the fact that leaf tissue is included as a component tissue (e.g. as a component of seedlings and whole plants) in several previous studies. On the other hand, it is interesting that there is only a single ABA-regulated gene, AT1G01470: *LEA14 (LATE EMBRYOGENESIS ABUNDANT 14) *found in common to all ABA transcriptome studies that we analyzed. This result hints that there might be greater flexibility in how tissues achieve a common end result of stress tolerance than in how they perform other functions such as primary metabolism.

Despite the lack of genes found in common to all studies, we nevertheless identified core ABA-regulated gene sets. Many of these core ABA-induced genes have already been assigned functional roles in response to ABA or another stress, or are members of a family (e.g. PP2C phosphatases [33-35]) already known to play such a role. In contrast, fewer ABA-repressed genes (Table 4) have known relationships to stress signaling or tolerance. Taken together with the observation that it has been more difficult to identify promoter motifs for ABA-repression than for ABA-induction, and with the fact that we had to reduce the number of experiments to 6 to identify a shared set of ~50 ABA-repressed genes, these results suggest a greater between-tissue diversity in the roles of ABA-repressed genes than those of ABA-induced genes. For example, our guard cell dataset includes 56 of the 67 core ABA-induced genes but only 24 of the 47 core ABA-repressed genes.

During drought stress, it is optimal for guard cells to lose water to promote stomatal closure while it is optimal for other cell types to retain water to maintain cellular hydration. We therefore expected to find some ABA-regulated genes unique to guard cells, and this expectation was satisfied. We identified ~150 ABA-induced and ~150 ABA-repressed genes in guard cells that were not identified as ABA-regulated genes in any of 11 previous studies of ABA-regulated transcriptomes (see Table S3 in Additional file 3). These genes represent potential guard cell-specific ABA-regulated genes. Consistent with this hypothesis, our predicted guard cell-specific motif GTCGG is significantly enriched in the upstream regions of these genes (*P *< 1.2E-04). Supporting the idea that these genes may have novel, as yet undescribed ABA-related functions in guard cells, many fewer genes in the lists of new and most significantly ABA-regulated guard cell genes (Tables 5, 6) are annotated as ABA- or stress-related than in the lists of core ABA-regulated genes (Tables 3, 4).

Additional file 4 provides the full list of the newly identified ABA-responsive genes of guard cells. Some of these genes already have been reported to be involved in stomatal movements or guard cell signaling. *JAR1 *(AT2G46370) is Jasmonate Resistant 1 and has been detected in guard cells [16]. This gene product has been reported to enhance ROS production and ABA sensitivity of guard cells [71] and its expression is ABA-induced in our guard cell data. Other hormone-regulated genes, such as the IAA inducible gene *IAA16 *(AT3G04730) and cytokinin response factor 3 *CRF3 *(AT5G53290), are also regulated by ABA in guard cells according to our microarray analysis. In addition, several Ca^2+^-related genes are identified as new ABA-induced genes in guard cells, such as *AtCAMBP25 *(AT2G41010) and *CBL2 *(AT5G55990). *CRY1/BLU1 *(AT4G08920) encodes the blue light photoreceptor cryptochrome 1 and has been reported to participate in blue light-regulation of stomatal movements [72], while NPH3 functions downstream of the PHOT1/PHOT2 blue light photoreceptors which mediate blue light-specific stomatal opening [73]. Our results indicate that these genes are ABA-repressed in guard cells, which might contribute to the phenomenon of ABA-repression of light-induced stomatal opening, particularly over a longer time scale. Sucrose is a significant osmoticum maintaining stomatal opening in the afternoon [74], and the sugar transporter STP1 has already been described as a guard cell-specific transporter that shows a peak in expression in the afternoon [75], in concert with the daily timing of sucrose accumulation into guard cells [74]. The observed repression of *STP1 *and the putative sugar transporter AT3G05160 by ABA might also contribute to long-term inhibition of stomatal opening.

### GO functional analysis

The GO category *lipid metabolic processes *is over-represented in ABA-induced genes of guard cells and leaves. Little is known concerning the relationship between ABA and lipid signaling in mesophyll cells; however, the guard cell result is consistent with functional evidence for roles of inositol phosphates (produced by the action of phospholipase C) and phosphatidic acid (produced by the action of phospholipase D) in ABA inhibition of stomatal opening [76-79]. In fact, among ABA-induced genes in this category we find AT5G58700, a phosphoinositide-specific phospholipase C family protein, and AT2G22240, an inositol-3-phosphate synthase, as well as two PLD isoforms: *PLDδ *(AT4G35790; also confirmed in our Q-PCR analysis) and *PLDζ *(AT3G05630), suggesting that activation of these genes may contribute toward long-term suppression of stomatal opening under drought conditions.

Conversely, several functional categories are underrepresented within the ABA-induced gene sets, including *translation*, *RNA binding*, *protein metabolism*, and *nucleobase metabolism*, consistent with the supposition that metabolism is generally down-regulated under stress conditions [80]. However, such down-regulation may be greater for leaves than for guard cells, given that GO categories associated with several types of metabolic processes are over-represented in leaf but not in guard cell ABA-repressed gene sets. This difference may reflect a requirement for guard cells to maintain metabolic activity and active stomatal regulation under drought conditions. One interesting observation is that the GO category *response to abiotic stimulus *is over-represented in both ABA-induced and ABA-repressed guard cell genes. Many of these genes are genes of known function in guard cells, and are discussed in the next section.

### Genes of known guard cell function, G protein regulation, and hormone cross-talk

Our analysis reveals that many genes which encode proteins with known functions in guard cell physiology are also ABA-regulated at the transcript level. This phenomenon was reported less robustly in a previous analysis [16] which queried only ~1/3 of the Arabidopsis genes assayed here. Changes in rates of solute transport resulting from non-transcription-related regulation of ion channels and transporters are central to rapid, osmotically-driven control of stomatal apertures [81,82]. However, our data show that many of the relevant transport proteins are regulated at the transcript level as well. Transcripts encoding the H^+ ^ATPase OST2, the inward K^+ ^channels KAT1 and KAT2, the sugar transporter STP1, the endosomal Na^+^/H^+ ^antiporter CHX20, and the nitrate importer CHL1 are all down-regulated in guard cells following ABA treatment, suggesting that their downregulation may be an important aspect of longer-duration inhibition of stomatal opening by ABA. Two known genes related to guard cell development, *FAMA *(*FMA*) which regulates proliferation of stomatal precursors as well as differentiation of guard mother cells [83], and *ATMPK3*, an environmentally responsive mitogen-activated protein kinase mediating stomatal development and patterning [84], are downregulated, as are several signaling proteins. Several other genes, functioning in the guard cell response to CO_2 _(*HT2*, *HIC*) and pathogens (e.g. *FLS2*), are slightly repressed by ABA (not shown), although failing to meet the threshold of significance used to categorize ABA-responsive genes in our microarray analyses.

ABA up-regulated genes include zeaxanthin epoxidase (ABA1), required for ABA synthesis. In addition, up-regulation of ABA-related transcription factors ABF3, ABF4, and AtMYB44 could provide positive feedback to the ABA response by promoting transcription of stress-related genes. A number of genes encoding known guard cell signaling proteins are up-regulated, including four protein phosphatase 2C's: PP2Ca, HAB1, ABI2, and ABI1. Since PP2C phosphatases are negative regulators of ABA signaling, their ABA-induced expression suggests instigation of negative feedback on the ABA response. This supposition is supported by the converse observation that five out of six genes encoding PYR/PYL/RCAR type soluble ABA receptors with documented function in guard cells [85-87] are down-regulated by ABA (out of the 14 receptors in PYR/PYL/RCAR family, only ten are represented on the ATH1 chip), as are two other genes encoding positive transducers of the ABA signal, the MAPK MPK3, and an enzyme involved in nitric oxide production, NIA2.

Analysis of T-DNA insertional mutants has implicated heterotrimeric G proteins in ABA responses of diverse tissues [58,59,88]. Mutant plants with G-protein subunit knockouts are ABA hyposensitive in aspects of guard cell ion channel and stomatal regulation [60,63,89,90] but, unexpectedly, ABA hypersensitive in inhibition of seed germination and root growth [61,91], indicating system-specificity of G-protein effectors. Interestingly, the leaf transcriptome actually has more genes co-regulated by ABA and G-proteins than the guard cell transcriptome. This result suggests important but as yet unidentified functions of ABA/G-protein co-regulation in mesophyll cell physiology. Conversely, in guard cells, this result implies that non-ABA related functions of G-proteins should be investigated. Indeed, based on phenotypes of *gpa1 *mutants, G-proteins also participate in CO_2 _and pathogen responses of guard cells [92,93].

Our results indicate that signaling cross-talk also occurs at the transcript level between ABA and other plant hormones. ABA-regulated and MJ-regulated genes identified by Nemhauser *et al. *[22] each have highly significant overlap with our ABA-regulated genes in both guard cells and leaves, and this is consistent with ABA-MJ crosstalk at the physiological level, in both guard cells [71,94-96] and other tissues [97]. In addition, a few genes designated as marker genes for hormones other than ABA in [22] are found to be significantly ABA-regulated in our transcriptomes. It is possible that the previous analysis [22] was confounded by the use of whole Arabidopsis seedlings as the material for microarray analysis, which may have masked hormonal co-regulation occurring in specific tissue or cell types. Although our evaluation of overlap is limited to one hormone (ABA), these results nevertheless indicate that the conclusion of [22], that plant hormones do not regulate common gene sets, merits re-assessment. Our work provides a starting point to re-assess core hormone regulatory modules and hormone marker genes.

## Conclusions

This microarray analysis investigates ABA regulation of gene expression in guard cells in a systematic genome-wide manner. A number of ABA-regulated genes of guard cells identified here overlap with ABA-regulated genes of other tissues while a subset of them show ABA-regulation unique to this cell type. A unique *cis*-acting motif, GTCGG, associated with ABA-induction of gene expression specifically in guard cells, was identified at the bioinformatic level. Many of the genes known to encode ion transporters associated with stomatal opening are down-regulated by ABA, providing one mechanism for long-term maintenance of stomatal closure during drought. We also found examples of both negative and positive feedback in the transcriptional regulation by ABA of known ABA-signaling genes, particularly with regard to the PYR/PYL/RCAR class of soluble ABA receptors and their downstream targets, the type 2C protein phosphatases. In conclusion, the results of this study engender new insights into the basic cell biology of guard cells, reveal common and unique elements of ABA-regulation of gene expression in guard cells, and set the stage for targeted biotechnological manipulations to improve plant water use efficiency.

## Methods

### Microarray hybridizations and Q-PCR

Our microarray data utilized the Affymetrix ATH1 chip and are from hybridizations described in detail in [31]. All of these data are available in the Gene Expression Omnibus (GEO) database http://www.ncbi.nlm.nih.gov/geo with accession no. GSE19520. To reprise, epidermal peels with guard cells as the only intact cell type were used as the source of RNA for our guard cell microarrays [31] (Figure 1A and Figure 1B), and leaves from the first and second rosette layer of five-week old plants were the RNA source for our leaf microarrays [31]. ABA treatments were 50 μM × 3 hr. and EtOH was used as the solvent control. If an epidermal peel preparation did not pass quality control parameters for guard cell purity it was not used for RNA isolation, and if an RNA preparation was not of high quality as indicated by Bioanalyzer profiling, it was discarded. Four genotypes were evaluated: Col (wild-type), *gpa1-4 *mutant, *agb1-2 *mutant, and *agb1-2 gpa1-4 *double mutant, where GPA1 (AT2G26300) and AGB1 (AT4G34460) are the Arabidopsis G protein α subunit and β subunit respectively. For each type of sample (guard cells or leaves), three independent biological replicates were obtained, resulting in a total of 48 microarray hybridizations (2 sample types × 4 genotypes × two treatments × 3 replicates).

For Q-PCR analysis, cDNA was aliquoted and kept at 4°C throughout each Q-PCR experiment to avoid discrepancy in the data because of freeze-thaw cycles. Real time PCR was performed using pre-mix containing SYBR-Green intercalating dye (BioRad). Actin was used as an internal control (*Actin 2*: AT3G18780 and *Actin 8*: AT1G49240) [98]. The positions of the oligonucleotide primers used for real-time PCR was chosen so that the size of all PCR products was between 100 and 150-bp. The suitability of the oligonucleotide sequences in terms of efficiency of annealing was evaluated in advance using the Primer 3 program. Q-PCR experiments were repeated thrice independently, and the data were averaged. The data obtained were analyzed with IQ5 software (Bio-Rad).

### Identification of differentially expressed genes

**Figure 11:**
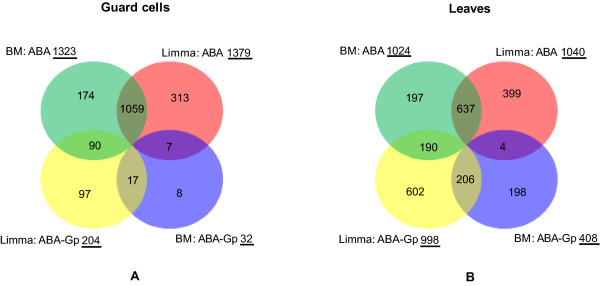
**Overlap distribution of ABA-responsive genes obtained by the two different methods**. (A) Guard cells; (B) Leaves. "BM: ABA" and "Limma: ABA" denote the G-protein-independent ABA-regulated genes obtained by the Boolean method and the linear model method, respectively. "BM: ABA-Gp" and "Limma: ABA-Gp" denote the G-protein-dependent ABA-regulated genes obtained by the Boolean method and the linear model method, respectively. The numbers with underlines are the total numbers of genes in the corresponding categories. For guard cells, the ABA-regulated gene set utilized here is the sum of the 1059 + 90 + 7 + 17 genes. For leaves, the ABA-regulated gene set utilized here is the sum of the 637 + 190 + 4 + 206 genes.

Our ABA-regulated gene sets consisted of the intersecting set of genes identified by two methods: a Boolean method and a linear model method (Figure 11).

In [31], we adopted a Boolean framework F(ABA,GPA1,AGB1) = B(ABA, A(GPA1, AGB1)) + C_ABA _to describe the regulatory modes of ABA and the G protein and identify ABA- and/or G protein-regulated genes. In this framework, GPA1 and AGB1 are Boolean variables which can have two states: 1 denoting 'on' (not knocked out) and 0 denoting 'off' (knocked out). ABA is also a Boolean variable: ABA = 1 indicates the presence of ABA (i.e. ABA treatment) and ABA = 0 represents the absence of ABA (i.e. solvent control). According to this framework, *B*_*4*_*(ABA, A) = ABA *and *B*_*13*_*(ABA,A) = not ABA *determine two Boolean rules describing ABA-regulation of gene expression independent of the heterotrimeric G protein. The rest of the Boolean rules B(ABA, A) describe G-protein-only regulation or the co-regulation of ABA and the G protein on gene expression. A correlation measure is used to assign differentially expressed genes to these Boolean rules [31]. In the present study, we used 1.5 as the correlation score threshold for ABA-only regulated genes to designate them as G-protein-independent ABA-regulated genes. This threshold controls the false discovery rate (FDR) within 0.001. In addition, those ABA-G-protein co-regulated genes that also exhibit greater than 2-fold expression change (with FDR less than 0.05) in response to ABA in the wild-type are designated as G-protein-dependent ABA regulated genes in the present study. In other words, all ABA-G-protein co-regulated genes described here satisfy the condition of showing ABA-regulation in the wild-type Col genotype.

To make the identification of differentially expressed genes with respect to ABA more reliable, we also used linear models combined with empirical Bayes methods to determine ABA-responsive genes [32]. This method is implemented by the limma package embedded in the Bioconductor project [99]. We followed the 2 × 4 factorial design process, using +ABA/-ABA as one factor and genotypes *agb1 gpa1 *mutant, *gpa1 *mutant, *agb1 *mutant, and wild-type as another factor. Contrasts of interest (ABA treated wild-type versus control wild-type, control mutants versus control wild-type, ABA treated mutants versus ABA treated wild-type) were extracted and the *P*-values for moderate *t*-tests were adjusted by Benjamini and Hochberg's method to control for the false discovery rate (FDR). We chose FDR <0.001 as a cut-off to select significantly differentially expressed genes. The *P*-value threshold for the contrast of ABA treated wild-type versus control wild-type was set at 0.0001, and the *P*-value threshold for the contrast of genotypes versus wild-type was set to 0.01. Those genes that are only significantly differentially expressed with respect to ABA are designated G-protein-independent ABA-regulated genes. Those genes that are significantly differentially expressed with respect to both ABA and genotypes are called G-protein-dependent ABA-regulated genes. As with the Boolean method, all genes designated as ABA-G-protein co-regulated from the linear model approach satisfy the condition of showing ABA-regulation in the wild-type Col genotype.

The Boolean method identified 1323 G-protein-independent ABA-regulated genes and 32 G-protein-dependent ABA-regulated genes in guard cells, with 1024 and 408 genes, respectively, identified in leaves. The linear model method identified 1379 G-protein-independent ABA regulated genes and 204 G-protein-dependent ABA-regulated genes in guard cells, with 1040 and 998 genes, determined in leaves. The G-protein-independent ABA-regulated genes determined by the two methods (intersection of green and red circles in Figure 11) and the ABA-regulated genes identified by both methods but with inconsistency in the existence of G-protein regulation (intersection of green and yellow circles plus intersection of red and blue circles in Figure 11) are designated G-protein-independent ABA-regulated genes in our final gene list. G-protein-dependent ABA-regulated genes that are confirmed by both methods (intersection of yellow and blue circles in Figure 11) are reported as G-protein-dependent ABA-regulated genes in our final gene list. The genes in each subsector of Figure 11A and Figure 11B are given in Additional file 7 and Additional file 8, respectively. The genes that are present in guard cells or leaves but not regulated by ABA at the transcript level (i.e. not present in any of the 8 subsectors of Figure 11A and Figure 11B) are given in Additional file 9 and Additional file 10, respectively.

### Promoter motif analysis

We performed a statistical analysis of the promoter sequences of ABA-responsive genes in guard cells and leaves. The 1000-bp upstream regions of all Arabidopsis gene sequences were obtained from the FTP site of the Arabidopsis Information Resource (TAIR) (TAIR9_upstream_1000_20090619.txt) [54]. Motif search was performed by writing custom Python scripts http://www.python.org which search a given motif pattern with an exact match without insert or mismatch. The significance of motif enrichment was computed by the hypergeometric cumulative distribution function. Specifically, when we examined the enrichment of a known *cis*-regulatory element, we counted the number of ABA-regulated genes in guard cells or leaves and the number of all genes in the chips whose promoter regions contain the *cis*-regulatory element. A *P*-value was obtained according to occurrences of this *cis*-regulatory element in ABA-regulated genes and in all genes. Positional distribution of a *cis*-regulatory element in ABA-regulated genes was obtained by shifting a window of the same length to scan the promoter region of each gene, and recording the occurrence positions. To identify possible new motifs that may be involved in ABA-regulated gene expression, we generated sets of 5-10mer sequences and calculated the enrichment significance of each sequence in our ABA-regulated gene sets. As there are many more significant motifs in ABA-induced genes than in ABA-repressed genes, we set 1.0E-10 and 1.0E-04 as the significance thresholds for new motifs of ABA-induction and ABA-repression respectively.

### Comparison of hormone-regulated gene sets

In the comparison of our ABA-regulated genes with previous ABA/hormone transcriptome studies, we used a representation factor (RF) and its associated *P*-value to evaluate whether the overlap of two gene sets from the same background is significant or not. The representation factor is defined by the ratio of the size of the real overlap to the expected number of common genes between the two gene sets:

where |*A*| is the number of genes in the gene set *A*, |*B*| is the number of genes in the gene set *B*, |*A*∩*B*| represents the number of genes common to gene set *A *and gene set *B*, and *N *is the total number of genes in the Arabidopsis genome (in this study, it is the number of genes covered by the Affymetrix ATH1 gene chip). The *P*-value associated with the representation factor was calculated by the hypergeometric distribution:

where *k *= |A∩B|, and the brackets indicate the binomial coefficient. Generally, the larger the representation factor is, the smaller the *P*-value, indicating that the overlap is more significant.

## Conflict of interests

The authors declare that they have no competing interests.

## Authors' contributions

SMA conceived the study, and participated in its design and coordination. SP, SL, TEG, and ZZ performed plant material preparation and mRNA extraction for microarray hybridizations. SP performed the Q-PCR experiments. RSW carried out all the computational analysis of the microarray data; SL participated in the initial stages of the analysis. RSW, RA and SMA analyzed the computational results, and drafted the manuscript. All authors commented on the manuscript and approved the final version.

## Supplementary Material

Additional file 1 **ABA-responsive genes in Arabidopsis guard cells**. This file lists the ABA-induced genes and ABA-repressed genes identified in Arabidopsis guard cells. Click here for file

Additional file 2 **ABA-responsive genes in Arabidopsis rosette leaves**. This file lists the ABA-induced genes and ABA-repressed genes identified in Arabidopsis rosette leaves. Click here for file

Additional file 3 **Supplementary materials of this study**. This file contains the supplementary materials for motif analysis and comparison of other ABA/hormone transcriptome studies. Click here for file

Additional file 4 **New ABA-responsive genes in Arabidopsis guard cells**. This file lists all the new ABA-responsive genes in guard cells not previously reported by any of the 11 previous ABA transcriptome studies that we evaluated. Click here for file

Additional file 5 **New ABA-responsive genes in Arabidopsis rosette leaves**. This file lists all the new ABA-responsive genes in leaves not previously reported by any of the 11 previous ABA transcriptome studies that we evaluated. Click here for file

Additional file 6 **Known guard cell signaling and development genes from published literature**. This file gives 149 guard cell signaling and development genes compiled from published literature which are classified as upregulated by ABA, down-regulated by ABA, and non-responsive to ABA at the transcript level. Click here for file

Additional file 7 **Genes identified as ABA-regulated in guard cells by each of the two methods**. This file lists the genes identified as ABA-regulated in guard cells by either the Boolean method, the linear model method, or both, i.e. the genes in each subsector of Figure 11A. Click here for file

Additional file 8 **Genes identified as ABA-regulated in leaves by each of the two methods**. This file lists the genes identified as ABA-regulated in leaves by either the Boolean method, the linear model method, or both, i.e. the genes in each subsector of Figure 11B. Click here for file

Additional file 9 **Genes present in guard cells but not regulated by ABA at the transcript level**. This file lists the genes that are present in guard cells but not regulated by ABA at the transcript level (i.e. not present in any of the 8 subsectors of Figure 11A). Click here for file

Additional file 10 **Genes present in leaves but not regulated by ABA at the transcript level**. This file lists the genes that are present in leaves but not regulated by ABA at the transcript level (i.e. not present in any of the 8 subsectors of Figure 11B). Click here for file
