# Supplementary material for: Common and unique elements of the ABA-regulated transcriptome of Arabidopsis guard cells
Source: BMC Genomics. 2011 May 9;12:216. doi: 10.1186/1471-2164-12-216 (PMC3115880; doi:10.1186/1471-2164-12-216)
Supplement: Additional file 3 — Supplementary materials of this study. This file contains the supplementary materials for motif analysis and comparison of other ABA/hormone transcriptome studies. [file 1471-2164-12-216-S3.PDF]

Common and unique elements of the ABA-regulated transcriptome of Arabidopsis guard cells

Supplementary Materials

1. Motif analysis of the promoter regions of ABA-regulated genes

To check whether those significant 5-mer motifs enriched in our gene sets are guard cell- or leaf-specific, we calculated the enrichment *P*-values of these motifs in published ABA-regulated gene sets using all genes in the whole genome as the background, and then calculated the enrichment of these motifs in our gene sets using published ABA-regulated gene sets as the background. The merged previously published ABA-regulated gene sets were from Hoth et al. (2002), Li et al. (2006), Xin et al. (2005), Matsui et al. (2008), Huang et al. (2007), Zeller et al. (2009), Sánchez et al. (2004), Nemhauser et al. (2006), Seki et al. (2002), and Okamoto et al. (2010). The results are shown in Table S1 and Table S2 respectively. Guard cell-specific or leaf-specific motifs are those that are significant in Table S2 but not significant in Table S1.

Table S1. The significance of 5-mer motifs in Table 2 in 1000-bp region upstream of previously published ABA-regulated genes. Motifs marked in bold are subsequences of ABRE considered in Table 1. Underlined motifs exhibit tissue specificity (GC vs LF) based on our analysis, and their *P*-values are highlighted in bold. [a]ABA-regulated genes from the previously published ABA studies. [b]ABA-regulated genes common to at least two studies.

**5-mer motifs**	ABA_up[a]	ABA_up[b]	**5-mer motifs**	ABA_down[a]	ABA_down[b]
**ACGTG**	2.3E-77	1.8E-85	CCACT	7.1E-20	4.4E-15
**CACGT**	7.2E-99	1.3E-105	CCAAC	3.3E-06	1.6E-07
CCACG	8.9E-30	5.3E-41	CAACT	5.2E-11	4.7E-08
ACACG	4.8E-67	6.4E-67	CACAT	5.9E-15	5.1E-07
**CGTGT**	2.4E-59	2.6E-73	GGTCC	2.1E-07	9.1E-10
**CGTGG**	2.0E-29	8.0E-19	<u>TGCAA</u>	**0.002**	**0.018**
**GTGTC**	3.9E-11	1.1E-13	GTCCC	1.1E-05	1.6E-05
GCCAC	4.2E-12	6.3E-14	GACCA	8.0E-09	1.3E-06
**GTGGC**	6.4E-06	8.7E-07			
GACAC	1.3E-12	2.8E-12			
**GACGT**	6.2E-10	3.3E-13			
CACGC	5.8E-14	7.1E-17			
**TACGT**	2.3E-33	3.4E-30			
<u>GTCGG</u>	**0.023**	**0.002**			
CCGAC	1.4E-06	1.0E-07			
ACGTA	9.6E-34	1.5E-32			
TCCAC	2.7E-13	3.9E-11			
TGTCG	6.3E-06	1.2E-06			

Table S2. The significance of 5-mer motifs in Table 2 in 1000-bp region upstream of guard cell or leaf ABA-regulated genes from our present microarray analysis, using previously published ABA-regulated gene sets as the background. Motifs marked in bold are subsequences of ABRE considered in

Table 1. Underlined motifs exhibit tissue specificity (GC vs LF) based on our analysis, and their *P*-values are highlighted in bold. *P*-values marked in light gray indicate that the motifs are not significant in the corresponding tissue (see Table 2). [a]The background is the ABA-regulated genes in the previously published ABA studies. [b]The background is those ABA-regulated genes common to at least two previously published ABA studies.

**5-mer motifs**	GC_up[a]	GC_up[b]	LF_up[a]	LF_up[b]	**5-mer motifs**	GC down[a]	GC down[b]	LF down[a]	LF down[b]
**ACGTG**	2.1E-52	9.7E-31	5.0E-12	0.008	CCACT	0.003	0.079	0.097	0.530
**CACGT**	1.8E-43	4.2E-21	7.9E-11	0.073	CCAAC	1.7E-04	0.006	0.019	0.192
CCACG	7.8E-38	6.0E-24	2.9E-04	0.543	CAACT	0.007	0.044	0.017	0.087
ACACG	3.9E-25	2.6E-12	1.6E-06	0.181	CACAT	0.028	0.044	0.110	0.169
**CGTGT**	1.2E-23	9.3E-09	3.5E-08	0.180	GGTCC	0.008	0.185	1.3E-04	0.011
**CGTGG**	2.1E-21	3.1E-20	0.008	0.096	<u>TGCAA</u>	0.016	0.019	**1.2E-04**	**1.0E-04**
**GTGTC**	5.4E-18	9.3E-14	0.152	0.824	GTCCC	0.016	0.089	0.001	0.009
GCCAC	5.0E-16	4.5E-12	0.651	0.991	GACCA	0.287	0.613	0.005	0.026
**GTGGC**	3.3E-13	1.3E-11	0.003	0.045					
GACAC	2.3E-09	9.6E-07	0.887	0.999					
**GACGT**	2.3E-08	7.5E-05	3.4E-04	0.052					
CACGC	4.0E-07	0.001	0.014	0.435					
**TACGT**	4.7E-04	0.201	2.0E-04	0.094					
<u>GTCGG</u>	**7.3E-11**	**3.6E-10**	0.024	0.115					
CCGAC	2.5E-08	2.8E-06	0.044	0.329					
ACGTA	9.4E-04	0.365	2.1E-05	0.046					
TCCAC	2.3E-06	1.3E-04	0.016	0.158					
TGTCG	6.8E-08	2.6E-06	0.089	0.413					

2. Comparison with other ABA transcriptome studies

Published ABA transcriptome experiments utilize different ABA treatments such as ABA concentration and treatment duration, different organs or tissue types, different platforms, different developmental stages, and different methods for identifying ABA-regulated genes. Table S3 gives a summary of experimental conditions in published ABA transcriptome studies (note: the ABA treatment duration is not documented in Hoth et al., 2002). E1 to E14 represent experiments from the following transcriptome studies: E1:Hoth et al. (2002), E2: Li et al. (2006), E3: Xin et al. (2005), E4: Matsui et al. (2008), E5: Huang et al. (2007), E6: Zeller et al. (2009), E7: Sánchez et al. (2004), E8: Nemhauser et al. (2006), E9: the guard cell ABA-regulated gene set identified in this study, E10: the leaf ABA-regulated gene set identified in this study, E11: Leonhardt et al. (2004) (guard cells), E12: Leonhardt et al. (2004) (mesophyll cells), E13: Seki et al. (2002), E14: Okamoto et al. (2010). The figures in the brackets in the first column represent the numbers of identified ABA-induced genes and ABA-repressed genes respectively.

Table S3: Summary of experimental conditions in published ABA transcriptome studies.

	**ABA dose**	**Treatment duration**	**Cell types**	**Age**	**Platform**	**Methods**
E1 (681, 717)	50 µM		Seedlings	4 weeks	MPSS	3-fold after a significance test is used. No FDR correction
E2 (692, 173)	10 µM	2h, 4h, 6h	Seedlings	7 days	Affy. ATH1	2.5-fold, no statistical test, no FDR correction
E3 (262, 125)	1 µM	4h	Seedlings	7 days	Affy. ATH1	2-fold after using Significance Analysis of

						Microarrays (SAM, Tusher et al, 2001) with FDR control
E4 (3623, 2729)	100 µM	2 h, 10 h	Whole plants	3 weeks	Tiling arrays	1.8 fold after Mann-Whitney U-test with FDR correction
E5 (915,849)	20 µM	6 h, 24 h	Seedlings, aerial parts	18 days	cDNA	SAM with FDR correction
E6 (1040,1194)	100 µM	1 h,10 h	Seedlings	10 days	Tiling arrays	Rank product, with FDR control
E7 (1490,1470)	50 µM	15 min	Lansberg *erecta* seedlings	3 weeks	Affy. ATH1	2-fold, no statistical test, no FDR correction
E8 (512,270)	10 µM	0.5 h, 1 h, 3 h	Seedlings	7 days	Affy. ATH1	Rank product and linear models, with FDR correction
E9 (696,477)	50 µM	3 h	Guard cells	5 weeks	Affy. ATH1	Boolean methods and linear models, with FDR correction (this study)
E10 (596,441)	50 µM	3 h	Rosette leaves	5 weeks	Affy. ATH1	Boolean methods and linear models, with FDR correction (this study)
E11 (87,65)	100 µM	4 h	Guard cells	5-6 weeks	Affy.AG	2-fold, no statistical test, no FDR correction
E12 (115,74)	100 µM	4 h	Mesophyll cells	5-6 weeks	Affy.AG	2-fold, no statistical test, no FDR correction
E13 (245, 34)	100 µM	1 h, 2 h, 5 h, 10 h, 24 h	Whole plants	3 weeks	cDNA	5-fold, no statistical test, no FDR correction
E14 (336, 586)	---	---	Imbibed seeds	Yellow-brown siliques	Tiling arrays	Mann-Whitney U-test with FDR correction

Different platforms cover different numbers of genes for transcript measurement. To make the pairwise comparison more reasonable, we incorporate that information into the overlap calculation. Seki et al. (2002) used a cDNA microarray which covers 6068 genes with AGI #. Hoth et al. (2001) used massively parallel signature sequencing (MPSS) to measure ABA-responsive gene expression. Since this technique does not give a fixed number of covered genes, we use the genes in the whole Arabidopsis genome released in 2002, which covers 27107 genes. Leonhardt et al. (2004) used the Affymetrix AG genechip which represents 7680 genes with AGI #. The Affymetrix ATH1 genechip which covers 22747 genes was used in Sánchez et al. (2007), Xin et al. (2005), Li et al. (2006), and Nemhauser et al. (2006). Huang et al. (2007) used a cDNA microarray from CyScribe Post-Labeling Kit (Amersham Biosciences) which represents 24064 genes. The Affymetrix Tiling array used in Matsui et al. (2008), Zeller et al. (2009), and Okamoto et al. (2010) covers 30751 genes with AGI #. When we calculate the overlap of two gene sets from two studies respectively, we only count those genes that are included in both platforms.

The percentage of genes common to two ABA-regulated gene sets *A* and *B* is calculated by

$$\text{Overlap}_{\text{per}}(A,B) = \frac{|A \cap B|}{|A \cup B|} \times 100 = \frac{|A \cap B|}{|A| + |B| - |A \cap B|} \times 100$$

where $|A|$ is the number of genes in the gene set A, $|B|$ is the number of genes in the gene set B, and $|A \cap B|$ represents the number of genes common to gene set A and gene set B. In addition, representation factor (RF), defined by the ratio of the real overlap to the expected number of common genes between the two gene sets (see Methods), is another way to characterize the overlap of two gene sets. It incorporates the total number of genes covered by a platform. Whether the overlap is significant or not can be evaluated by the hypergeometric distribution. An analogous method, Chi-square test, was used to test the significance of overlap between two hormone treatments in Nemhauser et al. (2006).

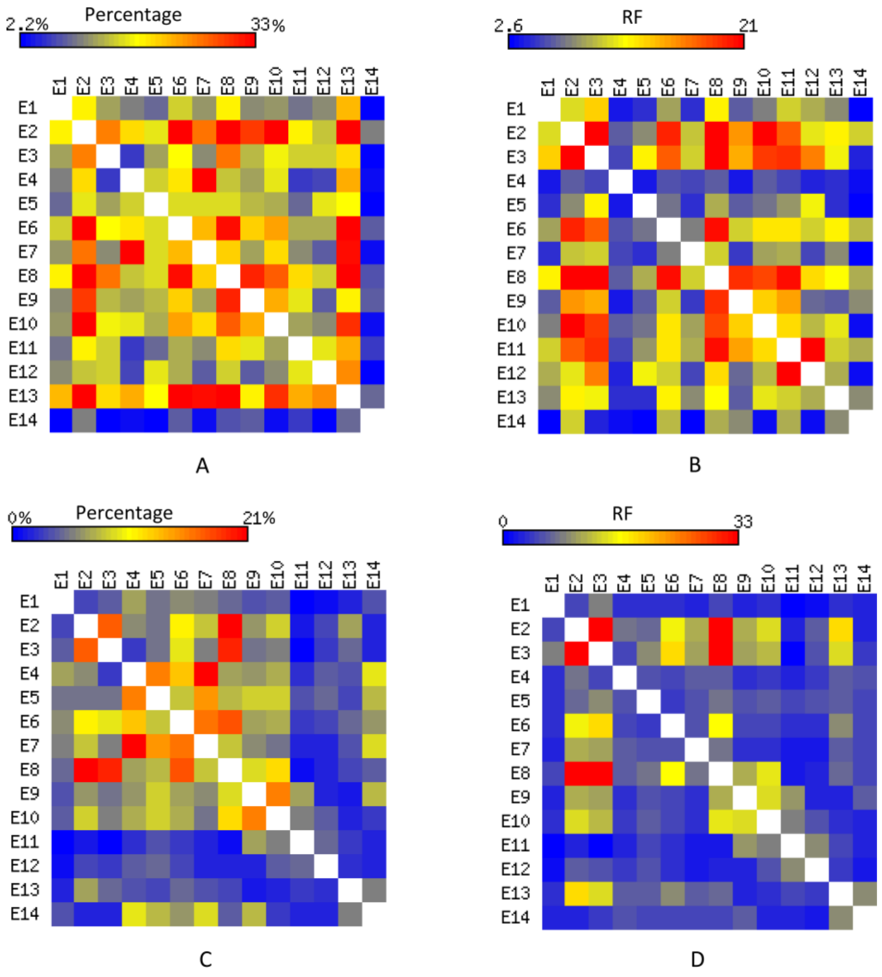

Figure S1. The pairwise overlaps of ABA-regulated genes identified in different studies. (A) Overlap percentage for ABA-induced genes. (B) Overlap representation factor for ABA-induced genes. (C) Overlap percentage for ABA-repressed genes. (D) Overlap representation factor for ABA-repressed genes.

Figure S1 shows the pairwise overlaps of ABA-regulated genes identified in different studies, characterized by percentrage and RF. From Figure S1(A), presumably due to the different experimental conditions, these studies of ABA regulation of gene expression do not have very high pairwise overlap percentages for ABA-induced genes. E14 (Okamoto et al., 2010) reports ABA responsive genes in imbibed seeds by measuring endogenous ABA levels in ABA-related mutants and generally has smallest overlap percentages with the ABA-induced genes in other studies. However, despite the fact that the overlap percentages are not very large, when compared with random overlaps,

all of the pairwise overlaps for ABA-induced genes are significant and their representation factors are shown in Figure S1(B). The overlap percentages for ABA-repressed genes are even smaller. Nevertheless, the *P*-values characterizing the significance of the overlaps show that, except for the ABA-repressed sets in Leonhardt et al. (2004) (E11-E12), Seki et al. (2002) (E13), and Okamoto et al. (2010) (E14), which have non-significant overlaps with several other studies, all other ABA-repressed gene sets have significant pairwise overlaps. The overlap representation factors comparing observed and expected numbers of common ABA-repressed genes are shown in Figure S1(D).

Nemhauser et al. (2006) performed an analysis of seven hormone-treated transcriptomes from 7-day-old Arabidopsis seedlings and identified target genes regulated by the hormones abscisic acid (ABA), gibberellic acid 3 (GA), indole-3-acetic acid (IAA, auxin), 1-amino-cyclopropane-1-carboxylic acid (ACC, ethylene precursor), zeatin (CK, cytokinin), brassinolide (BL, brassinosteroid), and methyl jasmonate (MJ, jasmonate). Six of these seven hormones regulate significant numbers of genes at the transcriptional level, identified by stringent analysis using two different methods (Nemhauser et al., 2006); the seventh hormone, GA, regulates only a few genes identified by a low-stringency linear model (Nemhauser et al., 2006). We took the intersection of the hormone-regulated gene sets identified by rank product and linear model methods (Nemhauser et al., 2006), and compared our ABA-regulated genes in guard cells and leaves with these hormonally regulated genes. The overlap of our ABA-regulated genes with hormone-regulated gene sets was calculated both in the same direction, that is, genes up- (down-) regulated by a hormone are compared with genes up- (down-) regulated by another hormone, and in the antagonistic direction. The comparison results in the same direction are given in Figure 11 in the main text. Table S4 summarizes the comparison in the antagonistic direction.

Table S4. Comparison of our ABA-regulated genes with hormone-regulated genes in Nemhauser et al. (2006) in an antagonistic way. Gray-shaded headers list numbers of genes regulated by each hormone, as observed in Nemhauser et al. (2006). Numbers in white boxes represent numbers of genes found in common with each gene set from Nemahuaser et al. (2006) and our guard cell and leaf ABA-regulated gene sets.

Hormone down-regulated genes	ABA (270 genes)	ACC (23 genes)	BL (33 genes)	IAA (61 genes)	MJ (231 genes)	CK (16 genes)
Guard cell ABA up-regulated genes (696 genes)	1	2	3	3	14	0
	AT2G39030	AT5G45950<br>AT3G56370	AT4G27260<br>AT3G06500<br>AT4G39800	AT2G39030<br>AT1G72510<br>AT2G23340	AT5G47610<br>AT5G11090<br>AT1G19860<br>AT5G20270<br>AT1G10740<br>AT5G37260<br>AT2G39700<br>AT5G59430<br>AT2G37750<br>AT2G36630<br>AT5G10930<br>AT5G45950<br>AT5G59350<br>AT5G01520	
Leaf ABA up-regulated genes (596 genes)	1	5	0	4	13	2
	AT2G39030	AT5G45950<br>AT5G14700<br>AT4G15620		AT2G39030<br>AT4G15630<br>AT2G38760	AT2G12290<br>AT3G63210<br>AT3G14560	AT5G43180<br>AT5G09440

		AT3G63210<br>AT4G15630		AT1G72510	AT2G33480<br>AT3G14280<br>AT5G20270<br>AT2G37750<br>AT1G63840<br>AT5G10930<br>AT5G45950<br>AT5G59350<br>AT1G48750<br>AT5G01520	
Hormone up-regulated genes	ABA<br>(512 genes)	ACC<br>(34 genes)	BL<br>(28 genes)	IAA<br>(198 genes)	MJ<br>(522 genes)	CK<br>(60 genes)
Guard cell ABA down-regulated genes<br>(477 genes)	2	4	0	10	12	1
	AT1G21400<br>AT4G28085	AT3G59900<br>AT2G44080<br>AT1G25560<br>AT2G40940		AT3G63440<br>AT5G51190<br>AT3G59900<br>AT5G12050<br>AT5G02760<br>AT4G17460<br>AT3G58120<br>AT4G37590<br>AT1G11000<br>AT2G41100	AT2G27690<br>AT4G17500<br>AT5G47220<br>AT3G23550<br>AT5G52120<br>AT1G06620<br>AT3G52870<br>AT5G43170<br>AT3G55130<br>AT2G36380<br>AT5G64560<br>AT5G14120	AT1G75450
Leaf ABA down-regulated genes<br>(441 genes)	0	0	1	8	7	3
			AT2G34510	AT3G63440<br>AT2G26710<br>AT5G04190<br>AT5G62280<br>AT5G02760<br>AT4G17460<br>AT1G70940<br>AT4G37590	AT2G27690<br>AT5G47220<br>AT3G23550<br>AT1G72940<br>AT4G31820<br>AT5G14120<br>AT2G40270	AT1G69040<br>AT2G34510<br>AT1G02370

In addition, Nemhauser et al. (2006) defined marker genes as those genes specifically regulated by one hormone in a high stringency analysis and not by any other hormone even in a lower stringency analysis. We compared these hormone marker genes with our ABA-regulated genes and found that some genes designated as marker genes for hormones other than ABA are actually regulated by ABA in our transcriptomes, as shown in Table S5 and Table S6.

Table S5. Comparison of our guard cell ABA-regulated genes with hormone-specific markers in Nemhauser et al. (2006). The first column gives numbers of marker genes for each hormone. The second column lists numbers of marker genes found in our guard cell ABA-regulated gene set. The AGI locus identifier and description of the genes are given in the third column and the fourth column respectively. Genes marked by asterisks are antagonistically regulated by the two hormones.

	No. of genes	AGI#	Description
ACC marker<br>(3 genes)	0		
BL marker	1	AT4G39800*	MI-1-P SYNTHASE (Myo-inositol-1-phosphate synthase)

(6 genes)			
IAA marker (57 genes)	7	AT1G11000*	MLO4 (MILDEW RESISTANCE LOCUS O 4)
		AT1G52830	IAA6 (indoleacetic acid-induced protein 6)
		AT2G23340*	AP2 domain-containing transcription factor
		AT3G15540	IAA19 (indoleacetic acid-induced protein 19)
		AT3G63440*	ATCKX6(CYTOKININ OXIDASE/DEHYDROGENASE 6)
		AT4G37590*	Phototropic-responsive NPH3 family protein
		AT5G51190*	AP2 domain-containing transcription factor
MJ marker (244 genes)	16	AT1G06620*	2-oxoglutarate-dependent dioxygenase
		AT1G67910	Similar to unknown protein (TAIR:AT1G24577.1)
		AT1G69850	ATNRT1:2 (NITRATE TRANSPORTER 1:2)
		AT2G04400	Indole-3-glycerol phosphate synthase (IGPS)
		AT2G27690*	CYP94C1 (cytochrome P450, family 94, subfamily C, polypeptide 1); oxygen binding
		AT3G02570	MEE31 (maternal effect embryo arrest 31)
		AT3G02875	ILR1 (IAA-LEUCINE RESISTANT 1); metallopeptidase
		AT3G22420	WNK2 (WITH NO K 2); kinase
		AT3G22740	HMT3 (Homocysteine S-methyltransferase 3)
		AT4G08170	Inositol 1,3,4-trisphosphate 5/6-kinase family protein
		AT4G17500*	ATERF-1 (ETHYLENE RESPONSIVE ELEMENT BINDING FACTOR 1)
		AT5G08520	Myb family transcription factor
		AT5G17490	RGL3 (RGA-LIKE 3); transcription factor
		AT5G52120*	ATPP2-A14 (Phloem protein 2-A14);
		AT5G52430	Hydroxyproline-rich glycoprotein family protein
		AT5G64560*	Magnesium transporter CorA-like family protein (MRS2-2)
CK marker (14 genes)	1	AT1G75450*	CKX5 (CYTOKININ OXIDASE 5); cytokinin dehydrogenase

Table S6. Comparison of our leaf ABA-regulated genes with hormone-specific markers in Nemhauser et al. (2006). The first column gives numbers of marker genes for each hormone. The second column lists numbers of marker genes found in our leaf ABA-regulated gene set. The AGI locus identifier and description of the genes are given in the third column and the fourth column respectively. Genes marked by asterisks are antagonistically regulated by the two hormones.

	No. of genes	AGI#	Description
ACC marker (3 genes)	0		
BL marker (6 genes)	0		
IAA marker (57 genes)	3	AT1G52830	IAA6 (indoleacetic acid-induced protein 6)
		AT3G63440*	ATCKX6(CYTOKININ OXIDASE/DEHYDROGENASE 6)
		AT4G37590*	Phototropic-responsive NPH3 family protein
MJ marker (244 genes)	20	AT1G17420	LOX3 (Lipoxygenase 3); iron ion binding
		AT1G28480	GRX480; thiol-disulfide exchange intermediate
		AT1G30135	JAZ8/TIFY5A (JASMONATE-ZIM-DOMAIN PROTEIN 8)

		AT1G61065	Similar to unknown protein AT4G27435.1
		AT1G72470	ATEXO70D1 (exocyst subunit EXO70 family protein D1)
		AT1G76070	Identical to uncharacterized protein At1g76070
		AT2G03980	GDSL-motif lipase/hydrolase family protein
		AT2G15760	calmodulin-binding protein
		AT2G27690*	CYP94C1 (cytochrome P450, family 94, subfamily C, polypeptide 1); oxygen binding
		AT2G33480*	ANAC041 (Arabidopsis NAC domain containing protein 41)
		AT2G34600	JAZ7/TIFY5B (JASMONATE-ZIM-DOMAIN PROTEIN 7)
		AT2G43510	ATTI1 (ARABIDOPSIS THALIANA TRYPSIN INHIBITOR PROTEIN 1)
		AT2G43520	ATTI2 (ARABIDOPSIS THALIANA TRYPSIN INHIBITOR PROTEIN 2)
		AT3G02875	ILR1 (IAA-LEUCINE RESISTANT 1); metallopeptidase
		AT3G55970	Oxidoreductase, 2OG-Fe(II) oxygenase family protein
		AT4G31820*	ENP (ENHANCER OF PINOID); signal transducer
		AT4G37010	caltractin, putative / centrin, putative
		AT4G37430	CYP91A2 (CYTOCHROME P450 MONOOXYGENASE 91A2); oxygen binding
		AT5G08520	myb family transcription factor
		AT5G67080	MAPKKK19 (Mitogen-activated protein kinase kinase kinase 19); kinase
CK marker (14 genes)	2	AT1G02370*	pentatricopeptide (PPR) repeat-containing protein
		AT5G43180*	Similar to unknown protein (TAIR:AT5G10580.1)

References

Hoth S, Morgante M, Sanchez JP, Hanafey MK, Tingey SV, Chua NH: **Genome-wide gene expression profiling in Arabidopsis thaliana reveals new targets of abscisic acid and largely impaired gene regulation in the abi1-1 mutant**. *J Cell Sci* 2002, **115**(Pt 24):4891-4900.

Huang D, Jaradat MR, Wu W, Ambrose SJ, Ross AR, Abrams SR, Cutler AJ: **Structural analogs of ABA reveal novel features of ABA perception and signaling in Arabidopsis**. *Plant J* 2007, **50**(3):414-428.

Leonhardt N, Kwak JM, Robert N, Waner D, Leonhardt G, Schroeder JI: **Microarray expression analyses of Arabidopsis guard cells and isolation of a recessive abscisic acid hypersensitive protein phosphatase 2C mutant**. *Plant Cell* 2004, **16**(3):596-615.

Li Y, Lee KK, Walsh S, Smith C, Hadingham S, Sorefan K, Cawley G, Bevan MW: **Establishing glucose- and ABA-regulated transcription networks in Arabidopsis by microarray analysis and promoter classification using a Relevance Vector Machine**. *Genome Res* 2006, **16**(3):414-427.

Matsui A, Ishida J, Morosawa T, Mochizuki Y, Kaminuma E, Endo TA, Okamoto M, Nambara E, Nakajima M, Kawashima M *et al*: **Arabidopsis transcriptome analysis under drought, cold, high-salinity and ABA treatment conditions using a tiling array**. *Plant Cell Physiol* 2008, **49**(8):1135-1149.

Nemhauser JL, Hong F, Chory J: **Different plant hormones regulate similar processes through largely nonoverlapping transcriptional responses**. *Cell* 2006, **126**(3):467-475.

Okamoto M, Tatematsu K, Matsui A, Morosawa T, Ishida J, Tanaka M, Endo TA, Mochizuki Y, Toyoda T,

Kamiya Y *et al*: **Genome-wide analysis of endogenous abscisic acid-mediated transcription in dry and imbibed seeds of Arabidopsis using tiling arrays**. *Plant J* 2010, **62**(1):39-51.

Sanchez JP, Duque P, Chua NH: **ABA activates ADPR cyclase and cADPR induces a subset of ABA-responsive genes in Arabidopsis**. *Plant J* 2004, **38**(3):381-395.

Seki M, Ishida J, Narusaka M, Fujita M, Nanjo T, Umezawa T, Kamiya A, Nakajima M, Enju A, Sakurai T *et al*: **Monitoring the expression pattern of around 7,000 Arabidopsis genes under ABA treatments using a full-length cDNA microarray**. *Funct Integr Genomics* 2002, **2**(6):282-291.

Tusher VG, Tibshirani R, Chu G: **Significance analysis of microarrays applied to the ionizing radiation response**. *Proc Natl Acad Sci U S A* 2001, **98**(9):5116-5121.

Xin Z, Zhao Y, Zheng ZL: **Transcriptome analysis reveals specific modulation of abscisic acid signaling by ROP10 small GTPase in Arabidopsis**. *Plant Physiol* 2005, **139**(3):1350-1365.

Zeller G, Henz SR, Widmer CK, Sachsenberg T, Ratsch G, Weigel D, Laubinger S: **Stress-induced changes in the Arabidopsis thaliana transcriptome analyzed using whole-genome tiling arrays**. *Plant J* 2009, **58**(6):1068-1082.
